# Implementation strategies to support fall prevention interventions in long-term care facilities for older persons: a systematic review

**DOI:** 10.1186/s12877-023-03738-z

**Published:** 2023-01-25

**Authors:** Neah Albasha, Leanne Ahern, Lauren O’Mahony, Ruth McCullagh, Nicola Cornally, Sheena McHugh, Suzanne Timmons

**Affiliations:** 1 Center for Gerontology and Rehabilitation, School of Medicine, University College Cork, St Finbarr’s Hospital, The Bungalow, Block 13, Douglas Road, Cork City, Ireland; 2 Rehabilitation Department, College of Health and Rehabilitation Sciences, Princess Nourah Bint Abdulrahman University, Riyadh, Saudi Arabia; 3 School of Clinical Therapies, University College Cork, Cork City, Ireland; 4 School of Nursing and Midwifery, University College Cork, Cork City, Ireland; 5 School of Public Health, University College Cork, Cork City, Ireland

**Keywords:** Falls prevention, Older person, Long-term care, Residential care, Implementation, Feasibility

## Abstract

**Background:**

Falls are common among older people in long-term care facilities (LTCFs). Falls cause considerable morbidity, mortality and reduced quality of life. Of numerous interventional studies of fall prevention interventions in LTCFs, some reduced falls. However, there are challenges to implementing these interventions in real-world (non-trial) clinical practice, and the implementation techniques may be crucial to successful translation. This systematic review thus aimed to synthesise the evidence on implementation strategies, implementation outcomes and clinical outcomes included in fall prevention intervention studies.

**Methods:**

A systematic search of six electronic databases (PubMed, CINAHL, EMBASE, PsycINFO, SCOPUS, Web of Science) and eight grey literature databases was conducted, involving papers published during 2001–2021, in English or Arabic, targeting original empirical studies of fall prevention interventions (experimental and quasi-experimental). Two seminal implementation frameworks guided the categorisation of implementation strategies and outcomes: the Expert Recommendations for Implementing Change (ERIC) Taxonomy and the Implementation Outcomes Framework. Four ERIC sub-categories and three additional implementation strategies were created to clarify overlapping definitions and reflect the implementation approach. Two independent researchers completed title/abstract and full-text screening, quality appraisal assessment, data abstraction and coding of the implementation strategies and outcomes. A narrative synthesis was performed to analyse results.

**Results:**

Four thousand three hundred ninety-seven potential papers were identified; 31 papers were included, describing 27 different fall prevention studies. These studies used 39 implementation strategies (3–17 per study). Educational and training strategies were used in almost all (*n* = 26), followed by evaluative strategies (*n* = 20) and developing stakeholders’ interrelationships (*n* = 20). Within educational and training strategies, *education outreach/meetings* (*n* = 17), *distributing educational materials* (*n* = 17) and *developing educational materials* (*n* = 13) were the most common, with 36 strategies coded to the ERIC taxonomy. Three strategies were added to allow coding of *once-off training, dynamic education* and *ongoing medical consultation.* Among the 15 studies reporting implementation outcomes, *fidelity* was the most common (*n* = 8).

**Conclusion:**

This is the first study to comprehensively identify the implementation strategies used in falls prevention interventions in LTCFs. Education is the most common implementation strategy used in this setting. This review highlighted that there was poor reporting of the implementation strategies, limited assessment of implementation outcomes, and there was no discernible pattern of implementation strategies used in effective interventions, which should be improved and clearly defined.

**Trial registration:**

This systematic review was registered on the PROSPERO database; registration number: CRD42021239604.

**Supplementary Information:**

The online version contains supplementary material available at 10.1186/s12877-023-03738-z.

## Background

Falling is a substantial health problem, as one of the most common geriatric syndromes among older people and linked to significant morbidity and mortality. The World Health Organisation (WHO) reports that approximately 30–50% of people aged 65 or above experience a fall at least annually, and 40% fall recurrently [1]. While falling is a problem in all health care settings, long-term care facilities (LTCFs) have the highest rate of falls of any setting – more than three times the rate of community settings, with an estimated 1.7 falls per resident-year [2, 3], and ranging between 3–13 falls per 1,000 bed days [4]. Indeed, half of residents in LTCFs experienced a fall more than once per year [5].

Falls often leads to physical and psychological consequences and economic burdens. One-third of residents suffer physical injuries after falling, most commonly hip fracture, estimated to occur in 3–5% annually [6]. Falls also produce psychological consequences such as depression, fear of falling, loss of confidence and decreased quality of life among residents [2, 7]. Falls in LTCFs are associated with a considerable economic burden to health care systems through prolonged hospitalisation. According to the National Institute for Clinical Excellence (NICE), the annual costs of falls and fall-related fractures are 2.3 and 1.7 billion pounds sterling, respectively [8]. Furthermore, fall-related death is considered the second most common cause of death globally [9]. The WHO (2021) estimates that 684,000 individuals die from falls each year, and it was reported in 2015 that 23% to 40% of fatal injuries in older people are due to falls [9, 10].

Falls are caused by various factors, including intrinsic factors, extrinsic factors and behaviour-related factors, and often result from a combination of factors. Intrinsic factors include chronic diseases, cognitive impairment, ageing, etc. Extrinsic factors include environmental hazards and medication, while behaviour-related factors linked to risk-taking and threat appraisal range from fear of falling on one side to risk-taking behaviour and impulsivity on the other [11–14]. Fall prevention interventions have been developed for LTCFs to identify and reduce risk factors related to falls and to reduce the rates of falls and fall-related injuries [5, 15]. They are typically multi-component interventions that provide standardised fall prevention interventions to reduce two or more risk factors related to falls. However, single intervention and multi-factorial interventions (i.e., where the intervention was tailored to the identified risk factors and needs of the residents) have also been described in the literature [16]. Interventions typically include exercises, staff education, environmental modification, medication review, etc.) [15, 16].

Many systematic reviews (SRs) and meta-analyses (MAs) have explored the effectiveness of fall prevention interventions in LTCFs [17–25] In 2015, Vlaeyen et al. published an SR and MA, reporting that fall prevention interventions overall reduced recurrent faller rates significantly, by 21%, but not the number of falls or fallers [18]. Within these studies, multifactorial interventions, as opposed to single or multiple interventions, appeared to reduce the number of fallers and recurrent fallers. Staff training as a single intervention appeared to increase the number of falls in two (low quality) studies. Lee et al., who conducted an MA in 2017, reported that exercise interventions reduced the fall rate substantially, and also decreased the number of falls and the fall rate when combined with other fall interventions [17]. Vitamin D supplementation also reduced the fall rate as a single intervention, according to a Cochrane collaborative review published in 2018, whereas other single or multifactorial interventions did not [23]. This review also noted the uncertainty of the effect of various fall prevention interventions in reducing the risk of falls. More recently, Gulka et al.’s SR and MA in 2020 indicated that all types of fall interventions reduced the numbers of falls (27%), fallers (20%), and recurrent fallers (30%) [22]. Exercise as a single intervention reduced the number of fallers (36%) and recurrent fallers (41%), respectively. However, it was reported that exercise interventions overall did not reduce falls, only exercise with a balance component, or using a technical device (like a balance board) or lasting over 6 months in duration [22]. It was also noted that staff education and training intervention revealed benefits in reducing falls and recurrent falls, unlike other single interventions [22].

Thus, the evidence for fall prevention interventions in LTCFs exist is mixed, reflecting the exact intervention in “single intervention” studies (with more evidence for exercise than staff education), the cohort studied (i.e. frailty and cognitive status influence outcomes), and the choice of outcome itself (e.g. falls, or fallers, or recurrent fallers). Clinicians work with older people who have varying levels of frailty, morbidity and functional capacity, and encounter many barriers to implementing fall prevention interventions in LTCFs [3, 26]. In 2017, an SR of eight mixed-methods studies found 27 barriers to implementing fall prevention interventions in LTCFs, with the most cited barriers as follows: staff feeling overwhelmed, helpless, frustrated and concerned about their ability to control falls management; staffing issues; limited knowledge and skills; and poor communication [27]. These challenges might impact on the success of fall prevention interventions, and thus it is necessary to identify techniques to facilitate the implementation of fall prevention interventions in LTCFs.

Implementation strategies are defined as “the methods and techniques used to enhance the adoption, implementation, and sustainability of a clinical programme or practice” [28]. Strategies such as audits and feedback, tailored strategies, educational meetings and educational outreach have been shown to improve the implementation of evidence-based care [29–32]. The successful implementation of fall prevention interventions can be achieved via multi-faceted influential strategies (e.g., audits and feedback, educational meetings, local opinion leaders) and strategies tailored to the needs of the clinical context, as determined by participants, that help staff, organisations and patients to overcome barriers and adopt a clinical intervention in real-world clinical settings [33]. The implementation strategies used, and how the implementation process is carried out (which is in turn assessed using implementation outcomes), may have an impact on the intervention’s effectiveness [34]. Nonetheless, previous SRs of fall prevention interventions in LTCFs have not included implementation strategies or outcomes. This review thus aims to synthesise the evidence on implementation strategies used to support fall prevention interventions in LTCFs, and also to describe the implementation outcomes included in the studies and how they were measured; along with clinical outcomes (i.e., Fall-related outcomes).

## Methods

A narrative SR was planned, given the nature of the topics of interest (implementation strategies and implementation outcomes) and the expected methodological and clinical heterogeneity of the studies. The SR was registered on the International Prospective Register of Systematic Reviews (PROSPERO) database (registration number CRD42021239604) and the protocol was published in BMJ Open [35]. The SR reporting followed the Preferred Reporting Items for Systematic Reviews and Meta-Analysis (PRISMA) guideline [36, 37].

### Eligibility criteria

All fall prevention intervention studies incorporating using experimental and quasi-experimental designs were included, considering single-site and cluster randomised controlled trials (RCTs), feasibility studies for RCTs (including pilot studies), pre and post-test design and quality improvement empirical studies. We only included qualitative studies, RCT protocol papers or mixed methods papers that accompanied eligible RCTs or pre-post studies, where these provided more detail on intervention implementation and clarified the context of implementation strategies (i.e., protocol papers). These complementary papers were not included in the quality appraisal process, but their content was used to aid our interpretation and synthesis of the main study. The inclusion and exclusion criteria were based on the Population, Intervention, Comparison, Outcome (PICO) framework, presented in Table 1. All relevant studies were published in English or Arabic (based on the authors’ native languages), between January 1, 2001, and December 31, 2021. The search dates reflected the relative newness of the field of implementation science such that no relevant data was expected prior to 2000.

**Table 1:** Inclusion and exclusion criteria according to the PICO framework

**Population**	**Inclusion:** • All staff members in LTCFs* working with older people (aged 65 and above) • Mixed population LTCFs were included where the intervention implementation in the older population was reported separately • Mixed settings (i.e., including staff in rehabilitation units) were included only if LTCF-related data was reported separately *LTCFs are defined by the WHO as follows: “Long-term care services include traditional health service such as management of chronic geriatric conditions, rehabilitation, palliation, promotion and preventative services that enable older people, who experience significant declines in capacity, to receive the care and support that allow them to live a life consistent with their basic rights, fundamental freedoms and human dignity” [38]; *this includes nursing homes and care homes* **Exclusion:** • The intervention was not directed at the staff of the LTCF • Studies included only individuals aged under 65, or had data that was not reported separately for the older people within a mixed-age population • Studies relating only to specific sub-populations in LTCFs (e.g., long-stay mental health residents, people with cognitive issues, intellectual disability, etc.) • Studies conducted outside of an LTCF
**Intervention**	**Inclusion:** Fall prevention interventions, whether a single-component or multifactorial/multicomponent intervention, where there was an implementation strategy or implementation process described **Exclusion:** Studies where the implementation strategy or process was not described
**Comparison**	**Inclusion**: Usual care or other interventions **Exclusion**:There was no restriction on the comparator used in eligible studies
**Outcomes**	**Inclusion/Exclusion**: The studies were not restricted based on the reported outcomes Our main focus was implementation outcomes (e.g., adoption, fidelity, etc.) [34]We also collated clinical outcomes in terms of the fall-related outcomes (noting that these were reported in various ways, such as fall risk reduction, fall rate reduction, time to first fall, occurrence of injurious falls, etc.) and staff-related outcomes

### Information sources and search strategy

After consulting with a medical librarian at University College Cork regarding search strategy terms and electronic and grey literature databases, a search was carried out on PubMed, CINAHL, EMBASE, Psyc INFO, SCOPUS and Web of Science for published intervention studies. In addition, a search was conducted for all published theses on OPEN GREY, Open Access Theses and Dissertations (OATD), ProQuest, British Library EThOS, EBSCO Open dissertation, RIAN, LENUS and CORA, to include studies where usable data existed in a published thesis but where paper publications were still in progress or there was a possibility of publication bias. The search was limited to the last 20 years, until 31^st^ December 2021. Search terms and medical subject headings (MeSH) incorporated keywords such as "long-term care facilities", "fall prevention" and "implementation outcomes", including free or controlled terms, combined with Boolean operators (see “Additional file 1 for search terms used, and PubMed database search strategy sample”). We conducted a forward and backward citation search of the studies included and a manual search for any related feasibility or implementation papers using the names of intervention study authors. We also hand-searched the reference lists of the published SRs on this topic.

### Selection process

Two reviewers (NA, LA) independently assessed the abstracts and titles of all papers retrieved to identify potentially relevant studies for a full review, using the evidence synthesis software Covidence (www.covidence.org). The software was used to eliminate the duplicate studies. Three independent reviewers (NA, LA and LO) then screened the full texts of all papers eligible for inclusion. All disagreements were resolved by a senior researcher (ST). In addition, two senior researchers (ST, NC) peer-reviewed and screened randomly selected papers to ensure the quality of the two screening phases: title/abstracts (100 checked) and full texts (50 checked). The consistency of all papers included was finally double-checked by a senior researcher (RM).

### Data collection process and data items

Two independent reviewers (NA, LA) extracted data from papers included. The data were compared, and any disagreement was resolved by discussing until consensus was reached. The extraction table included the following details: first author, publication year, country, study design, study duration (intervention and follow-up periods), participant eligibility criteria and sample size (e.g., patient criteria, staff criteria), participant data, fall intervention characteristics (e.g., type of intervention, usual care or control intervention), implementation strategy, implementation outcomes (e.g., fidelity) and intervention outcome (i.e., direct fall-related outcomes).

The outcomes (or outcome domains) of the present review were as follows. Firstly, the implementation strategies of fall prevention interventions were categorised and labelled using the Expert Recommendation for Implementing Change taxonomy (ERIC) [39, 40]. This framework provides a comprehensive definition of 73 discrete implementation strategies, mapped under nine subheadings. This list was developed by researchers and expert clinicians, generating expert consensus on a common set of terms and definitions, refining the original compilation implementation strategy list of Powell et al. (2009) from health care and mental health care literature [41]. The descriptions of the implementation strategies in many studies can be varied, leading to difficulty in comparing and categorising them; selecting ERIC in this review facilitated a more systematic description and reporting of the implementation strategies, regardless of terminology discrepancies.

Secondly, the implementation outcomes were categorised based on the Proctor et al. taxonomy, which defines eight implementation outcomes: feasibility, fidelity, adoption, appropriateness, implementation cost, sustainability, acceptability and penetration [34, 42]. An expert group from the implementation sciences developed the implementation outcome taxonomy to identify the precise concept for labelling the implementation process by collating definitions of implementation outcomes and determining the distinctions between them.

The clinical outcome of interest was the effect of the intervention on fall reduction, which was reported in different ways in intervention studies, in terms of fall risk reduction, fall rate reduction, time to first fall, etc. We considered the primary outcome without distinguishing between injurious and non-injurious falls, and secondary outcomes (e.g., mortality rate) are not presented in this review.

### Risk of bias assessment

Two independent reviewers (NA, LA) assessed the quality of the papers included using the relevant checklists for RCT and quasi-experimental studies from the Joanna Briggs Institute (JBI) Critical Appraisal Tool, in which these checklists assessed studies according to a total of 13 and nine assessment criteria, respectively [43, 44]. All disagreements were resolved by discussion. Each item was rated as "yes", "no", "unclear" or "not applicable", based on whether or not the information was obviously reported or if the criteria were irrelevant to the study [45]. One point was given to every criterion rated "yes", whereas 0 was given to criteria rated "no", "unclear" or "not applicable". Following this, a total score for each study was calculated by adding all of the “yes” responses for each study out of 13 criteria for RCT and nine criteria for quasi-experimental studies. The authors a priori decided to not exclude papers based on their quality appraisal results, so as to be able to include quasi-experimental and quality improvement studies which were likely to have useful implementation data.

### Synthesis methods

A narrative synthesis was undertaken because of the methodological and clinical heterogeneity of the studies included in this review (both variety in intervention components and outcome measures), and a metanalysis was not practical. Two independent reviewers (NA, LA) coded each study's implementation strategies data according to the best match with the labels and definitions for the 73 ERIC strategies, then synthesised them into the nine subheadings. To code strategies we used the definitions in the published ERIC taxonomy and the detailed descriptions contained in the supplementary files of that paper. We used NVivo software (QSR International) to organise the data for the coding and labelling process, and we resolved any conflict via discussion. There was a challenge in coding some education-related text to ERIC strategies, where the described strategy did not fit into an existing ERIC strategy. As a result, additional strategies, in terms of frequency, mode of delivery, variety of information delivery techniques, and consultation-focused strategies, were required. Similarly, the implementation outcomes were coded and synthesised using Proctor’s taxonomy, considering the actual measurement of their outcome and data reporting from the target population. A senior researcher (SM) checked all implementation strategy and outcome coding.

### Coding assumptions

When coding implementation strategies, we made the following assumptions: firstly, all references to the assessment of environment hazards and modification, such as rugs, slippery floors, electrical cords, floor lighting at night, etc., or the assessment/repair of assistive devices, when provided as a core component of the intervention, were excluded from coding, because these were considered clinical interventions for preventing falls. Although we excluded environmental modifications because they were clinical interventions for preventing falls, we did include infrastructure changes at the organisation level, because they support the implementation of intervention strategies. Secondly, any references to educating residents as part of the intervention were not coded, despite being similar to the “prepare patients to be active" implementation strategy, because this is a part of a fall prevention intervention, not an implementation intervention. Thirdly, we used the ERIC label “create a learning collaborative” for studies where there were efforts to bring together the intervention providers, although the collaboration purpose was not always clear. Fourthly, we categorised references to staff reminders as “clinicians’ reminders” strategies, without detailed description, because such information was not stated in the paper. This assumption was made to ensure that even the minimum level of content of any strategies was acknowledged. In addition, any reference to individuals who assisted staff in their practice by providing problem-solving, discussion or support, whether involving internal or external facilitators, was coded under the “facilitation” strategy.

Some strategies involved both audit and feedback, whereas other interventions only implemented audit; we grouped these together, noting which aspect was used. Similarly, the development and the distribution of educational material were deemed likely to occur together, and so were grouped together, unless the study explicitly described using existing educational material sourced from elsewhere. Furthermore, the definition of the strategy “conduct educational outreach visits” contained many aspects of teaching delivery and was broad in scope based on the detailed description in the ERIC supplementary file. To more accurately capture the nature of the strategy, we adapted four subcategories of an existing strategy (i.e., education meeting/outreach) based on the mode of delivery (e.g., in-service vs online) and frequency of education/training delivery (e.g., once off vs ongoing sessions). When using a variety of information delivery methods (i.e., dynamic training versus dynamic education), a distinction has been made between training (e.g., group discussion) and education (e.g., videos, posters). We distinguished references to “ongoing consultation strategies” (which provided consultations focused on fall prevention implementation strategies) from references to medical consultation to solve medical problems linked to falls. Thus, three additional strategies have been identified under one of the ERIC subheadings, namely training and educating stakeholders. All new subcategories and strategies developed in this SR that were not found in ERIC lists were added to the codebook (see “Additional file 4”).

### Reporting bias assessment

We contacted the authors of studies where only the abstracts or trial protocols were published, to seek full-text articles and we included published theses to avoid potential publication bias for unpublished negative studies or slow publication of thesis results.

## Result

### Study selection

**Fig. 1:**
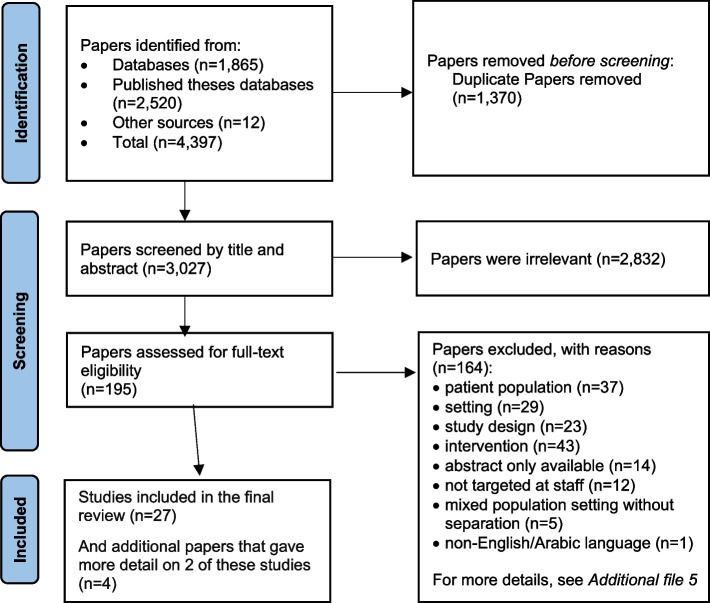
PRISMA flow diagram of study selection and inclusion process. From: Page MJ, McKenzie JE, Bossuyt PM, Boutron I, Hoffmann TC, Mulrow CD, et al. The PRISMA 2020 statement: an updated guideline for reporting systematic reviews. BMJ 2021;372:n71. https://doi.org/10.1136/bmj.n71

We identified 4,397 papers from the search of all databases and other resources. After duplicate studies were removed, 3,027 unique papers remained for title/abstract screening. Of these, 2,832 papers were irrelevant and excluded based on titles/abstracts, leaving 195 papers for full-text screening. In total, 27 studies met our eligibility criteria and were included [46–72], and four additional papers related to two of the intervention studies were included to provide more detail on the intervention implementation [73–76] (See Fig. 1).

### Study characteristics

Overall, 7 out of 27 studies included were cluster randomised controlled trials (CRCTs) [60–66]. The remaining studies were quasi-experimental studies, 14 of which were peer-reviewed journal articles [46–59] and six of which were published theses [67–72]. A pre-post design accounted for the majority of the quasi-experimental studies, and nine of these were quality improvement projects. Additionally, two out of 4 additional papers were protocol papers [74, 76], while the other two referred to a single intervention. In the first, the validated process of fall outcomes was measured using clinical vignettes and chart abstraction in a cross-sectional study within CRCT [75]. The second was a qualitative study comparing staff descriptions of the learning climate, the use of social constructivist learning processes and outcomes between the intervention and control groups [73].

In total, 17 studies were conducted in the United States; six were in European countries (Spain, Poland, Sweden, Belgium, Germany and Scotland) [47, 51, 54, 60, 63, 66]; three were in Australia or New Zealand [56, 61, 65], while one study was conducted in Japan [58]. Overall, 10 out of 27 studies were conducted in nursing homes [47, 49–52, 54, 59, 63, 64, 66]; four involved skilled nursing facilities [57, 67, 69, 71]; one was in a state-provided veterans home [55]. In total, 16 were carried out at single sites [46–48, 50, 53, 54, 56, 58, 59, 67–72, 77]. In eleven studies, the intervention was directed at nursing staff only (i.e., registered nurses, certified nurses’ assistants, licensed practical nurses) [46, 48, 56, 58, 63, 66–71]. Nine studies delivered a single fall-prevention intervention (e.g., staff education) [48, 52, 62, 63, 65, 67, 68, 70, 71], while nine included multi-component interventions (i.e., a standardised approach for all participants) [46, 49, 50, 55, 59, 64, 66, 69, 72], and nine were multi-factorial interventions that tailored the intervention to the needs of the participants and the residents [47, 51, 53, 54, 56, 60, 61, 77]. The majority of intervention components concentrated on training and educating staff in falls risk assessment, risk factor modification, and post-fall management and medication review (see Table 2). The duration of the included interventions ranged from four to 104 weeks, and most (21 studies) involved falls interventions lasting 26 weeks or less. Only eleven studies included follow-up periods after the intervention, ranging from 13 to 52 weeks. Ten studies involved control groups, eight of which received usual care, while two studies provided interventions to control groups (Table 2).

**Table 2:** Study characteristics

First author, publication year, country	Study design, setting	Participant eligibility criteria, sample size (n)	Falls intervention characteristics					Intervention outcomes (primary)	
			Type	Intervention components	Baseline assessment of staff and/or facilities	Duration (weeks)	Control	Clinical outcome/ Fall-related outcome	Staff-related outcome
Bonner 2007, US [46]	Pre-post-test, LTCF	•Single-site facility •Staff: all staff (178): 8 nurses; 40 CNAs; 20 non-licensed personnel; total *n* = 68 •Residents: N/R	MCI	•Educational intervention •Falls champion •Weekly interdisciplinary rounds	•10-item knowledge test developed by investigators • Fall rates (a month prior)	•I: 9 wks • F: None	N/A	**Fall rates** (falls/100 residents per month)**:** Pre-test: 16.1%. At 30 days: 12.3%. At 60 days: 9%	**Staff knowledge test:** pre-test score of 86.78%; 60-day mean post-test score of 90.69%
Gama 2011, Spain [47]	QI (pre-post-test), NH	•Single-site facility •Residents: those who could walk 5 m independently; *n* = 127 •Staff: “staff of the institution” (*n*: N/R)	MFI	QI cycle including: •Local building & piloting of quality criteria (4 structural and 9 process) •Specific interventions involving educational activities; Change process and record systems	•13 criteria for quality of fall prevention were assessed •Fall occurrences: in a 60 randomly selected residents (recorded 6 months prior)	•I & F: 26 wks	N/A	**Fall numbers:** 53 falls in a year; pre-intervention: 32, post-intervention: 21	
Leverenz, 2018, US [48]	Pre-post-test pilot study, LTCF	•Single-site facility •Staff: Full-time nursing staff who completed the education session; *n* = 8 (6 CNAs; 1 LPN; 1 RN) •Residents: N/A	SI	•Staff education & training course with 6 LTCF learning modules/fall prevention content areas: vision; assistive devices; environment; pressure and motion alarm; mobility; therapeutic use-of-self	Staff self-efficacy using SEPF-A and SEPF-N scales	•I: 5 wks • F: None	N/A	N/A	•**SEPF-N** cumulative average score (*n* = 6/8): pre-training: range 18 to 48; post-training: range 31 to 48 •**SEPF-A** cumulative average score (*n* = 2/8): pre-training: range 19 to 24; post-training: range 34 to 48
Rask., 2007, US [49]	QI, NHs	19 NHs (initially 9; plus 10 after 4 months); 23 control NHs •Staff: FMP team involved a PT or OT, 2–4 nursing assistants, a maintenance member and a director of nursing (n: N/R) •Residents: all residents of participating NHs (*n*: N/R)	MCI	•Organisational leadership buy-in and support •Facility preparation-based falls coordinator and MDT •Intensive training and education •Ongoing consultation & oversight by advanced nurse experts in FMP team	Data on falls from MyInnerView quality improvement software in Georgia that enabled the examination of data for intervention and non-intervention facilities	•I & F: 26 wks	Usual care	•**Fall rates** (falls/100 residents per month): IG: stable (17.3 to 16.4); CG: increased 26% from 15.0 to 18.9 **•The level of physical restraint**: decreased in both groups; IG: 7.9% to 4.4%; CG: 7.0% to 4.9%	
Jackson, 2016, US [50]	QI (pre-post-test), NH	•Single-site facility, 150 beds in a suburban area •Residents: all except dementia unit; *n* = 123 •Staff: convenience sample of 10 staff (4 nurses, 4 nurse aides, 2 therapists) who provided direct patient care (employed full-time for at least six months at NH)	MCI	Included recommendations of the Agency of Healthcare Quality and Research (AHRQ) •Mobility training conducted by PT for all staff and FT •Hourly staff rounding on residents • Physical therapy: Residents encouraged to participate in activities outside of their rooms •Post-fall assessment	•Staff knowledge questionnaire from AHRQ •Data abstraction of fall characteristics	•I: 17 wks • F: None	N/A	•**Fall rates:** Mean monthly scores pre-I (24.5), post-2-month (13.5), post-4 -months (9.5; 54% decrease in 4 months) •BRIGGS fall risk assessments (newly admitted residents; duration: 4 months): 76% had a high risk of falls •**Fall characteristics:** 54% occurred by day, 37% evening and 9% at night. 60% fell in own rooms, 28% in dayroom, 4% in dining room, 4% in hallway •42% had fall-related injuries, of which 11% fractures, 21% skin tears, 16% lacerations and 53% haematomas	**Staff knowledge test:** The average score on the pre-test was 74, and after 2 months, it was 90; after 4-month post-testing, it was 92
Szczerbinksa, 2010, Poland [51]	Pre-post-test, NHs	•3 NHs •Residents: all residents of NHs unless unable to walk or cognitive/behavioural issues or medical CI to exercise. NH A: *n* = 94 residents; NH B: *n* = 88, NH C: *n* = 40; total *n* = 222 •Staff: all staff in NHs (nurses, care assistants, PT); *n* = N/R	MFI	EUNESE intervention: broad staff involvement in falls risk assessment and referral to exercise programme •Staff training •Exercise intervention •Nurses and care assistant staff conducted the intervention in NHs A and B, PTs in NH C	•The percentage of residents able to walk (assisted or unassisted) •The ratio of staff to residents •The safety index against falls	•I: 52 wks •F: 26 wks	N/A	**•Fall incidence:** 144 falls: NHA = 53, NHB = 60, NHC = 31 •**Falls per month per 100 residents**: NHA = 53.5, NHB = 45.8, NHC = 67.4 **•Falls incidence medians (pre- vs implementation vs follow-up):** NHA: 5.05 vs 2.52 vs 0.50; H = 8.84, *p* < 0.05 NHB: 4.58 vs 0.38 vs 1.90; H = 8.52, *p* < 0.05 NHC:3.26 vs 5.43 vs 1.08; H = 3.94, ns	
Colon-Emeric, ^A^ 2006, US [52]	QI (pre-post-test), NHs	•36 NHs; 353 non-intervention NHs •Staff: 2–3 per facility (nursing, administration, PT, pharmacy) (*n* = unknown) •Residents: residents in each facility (*n* = 832)	SI	•Staff education and monthly teleconferences using Change Package emphasised fall-risk screening, labelling and risk-factor reduction	Chart abstraction of falls data (IG only)	•I: 39 wks •F: None	Usual care	•**Fall rates:** IG: pre: 18.2%; post: 15.4%; CG: pre: 12.3%; post: 11% (*p* = .56) **Fall rates** (per 1,000 resident-days): ***Self-report data:*** 6.1 to 5.6 falls; *p* = .31; ***Chart abstraction***: 28.6% to 37.5%, *p* = .17 •Falls screening increased from 51 to 68% (*P* < .01); Vitamin D prescriptions 40% to 48%, *P* < .05; use of sedative-hypnotics 19% to 12%, *P* < .01	
Wongrakpanich, 2018, US [53]	QI (pre-post-test), LTCF	•Single-site facility •Resident participants: all aged 65 + , residing in the facility; no exclusion criteria (*n* = 32) •Staff participants: all staff participated (PTs, geriatricians and RNs participated) (*n* = 12)	MFI	“STOP-FALLING” checklist included: •Vitamin D supplementation •Patient & family education •Orthostatic vital signs •Physical therapy •Hearing aids and evaluation •Medication review •IN-room safety evaluation •Glasses and vision evaluation	Fall rates, the number of fall-related injuries (minor and major) and recurrent falls (3 months prior)	•I: 13 wks •F: None	N/A	•**No. of falls:** pre: 22 fallers; post: 13 **•Fall-related injuries:** pre: 13 (1 major, 12 minor); post: 8 (minor: 8, major: 0) **•Fall rate:** 2.80 to 1.65 falls/person-year **•No. frequent fallers:** 5.00 to 2.30/mo.; *P* < .001, 95% CI 1.78–3.56 **•No. falls without injuries:** (3.00 to 1.67/mo.; *P* < .001, 95% CI 0.69- 1.97), **•No. minor injuries** (4.00 to 2.67/mo.; P < .015, 95% CI 0.14- 2.52) **•No. major injuries** (0.33 to 0.00/mo.; P < .001, 95% CI 0.13–0.53)	
Cooper, 2017, Scotland [54]	QI, NH	•Single-site facility •Residents: newly admitted (*n* = 29) •Staffs: “staff in facility” (*n* = 31)	MFI	•Staff training- implement/test tool, reflect on changes; ‘driver’ diagram •Fall champions •Fall leadership •Family involvement •Post-fall huddles	•Fall rate and characteristics (3 months prior)	•I: 26 wks •F: None	N/A	**• Fall rate** (per 1,000 occupied bed days): Mean: 49 to 23, demonstrating an improvement of 36.3%	
Zubkoff, 2019, US [55]	QI, SVH	•26 SVHs •Residents: elderly, 96% male; (*n* = unknown) •Staff: Falls teams in each facility: team leader; senior-level support person; nurse, physician or nurse practitioner champion; physical therapist pharmacist; (*n* = N/R)	MCI	‘Virtual breakthrough series’ (VBTS) model (27 interventions) with •Webinar for staff education •Open discussion sessions •Coaching •Mentoring •Fall champion •Leadership	•Baseline report of the MDT, planned aims and current fall prevention efforts •Fall rate (prior 6 months)	•I: 26 wks •F: 26 wks	N/A	(5 sites excluded as submitted less than 50% of the monthly data) •**Fall rates** (/100 days): pre: 28.5; during: 29.2 (*p* > 0.238); after: 27.5 (*p* > 0.136) •**Fall injury rate** (/100 days): pre: 7.4, during: 6.6 (*p* = 0.009); after: 5.6 (*p* = 0.005) •**Minor injury rate** (/100 census days): pre: 6.4; after: 5.8 (*p* = 0.000)	
Beasley, 2009, Australia [56]	QI, LTCF	•56-bed single-site catering for high- & low-care, incorporating a 6-bed secure dementia unit •Residents: all at medium/high fall risk on admission (*n* = 20) •Staff: Enrolled nurses & care staff on duty over 3 days *(n* = 20)	MFI	•Audit and feedback process using a PACES-JBI software programme •Practice standards, evidence-based strategies (education intervention for staff and residents, medication review, environmental assessment, fall risk assessment)	Baseline audit: •Staff attendance at fall prevention training 12 months prior •Falls data	•I: 20 wks •F: None	N/A	•**No. falls per month:** pre-intervention: 21–23 post-intervention: 12	
Hofmann 2003, US [59]	Pre-post-test; NH	•Single-site (not-for-profit) •Residents: older people, 55% aged over 85 years (*n* = 120) •Staff: multiple disciplines from the facility (*n*: N/R)	MCI	•Environmental actions •Incorporating additional staff through shift changes •Restorative Activity Programme	• Fall rate: fall incidence from medical records (a year prior)	•I: 52 wks •F: 52 wks	N/A	•**No. falls:** pre: 479 falls; post: 299 falls; total reduction: 38% (*P* = 0.0003) •**No. & rate of fractures**: pre: 16, 3.3% fracture rate; post: 8, 2.7% fracture rate, total reduction: 50% **•Fall rates by shift:** 1. 7AM-3PM shift: pre: 167; post: 155 2. 3-11PM: pre: 221; post: 115; 63% had fractures 3. 11PM-7AM: pre: 91; post: 29 **•Recurrent fallers:** pre: N/A; post: 13 individuals (range: 2–9 falls); 20% of total no. of falls	
Theodos, 2004, US [57, 76]	Pre-post-test, SNF	•Single-site, 156-bed skilled nursing home •Residents: long-stay only (*n* = 145) •Staff: ‘all staff at facility (*n*: N/R)	MFI	•Staff training •Post-fall assessment •Case management intervention for fall-risk residents •Exercise •Bed transfer	•Fall incidence (27 weeks prior)	•I: 27 wks •F: None	N/A	•**No. falls**: pre: 207 falls; post: 173 falls •**Av. weekly census**: pre: 137.4 (0.0609 falls/resident/week); post: 141.34 (0.0473 falls/resident/week) •**Fall rate**: pre: Mean: 0.060, SD: 0.022; post: Mean: 0.047, SD: 0.026; paired t-test: *p* = 0.0486; Chi-Square test: p = 0.009 •**Fall occurrence by shift**: evening (51%); day (31%); night (18%) •**Fall occurrence details**: ambulating (70%), 25% fell from chair, wheelchair or commode; 5% due to ‘various causes ‘	
Kato, 2008, Japanese [58]	Action research, pre-post-test, LTCF	•Two wards in a single-site •Residents: older (IG: 31; CG: 20) •Staff: nurses and caregiving staff members; (IG: 14; CG: 10)	MFI	•Staff education •Assessment of individual risks • Care adapted to risks •Consultation about fall-related problems •Modification of care when falls	•The number of falls and injuries (6 months prior) •The Generalized Self-Efficacy Scale and the Social Support Scale for staff	•I: 26 wks •F: None	Usual care	•**No. falls**: IG: pre: 37; post: 27; CG: unchanged •**Fall rate per 1,000 residential days**: IG: 7.6 to 5.0; CG: 4.8 to 4.3 **•No. fallers**: IG: 11 (35.5%) to 14 (45.2%); CG: 6 (30.0%) to 7 (35.0%) **•No. injuries:** IG: reduced from 13 (41.9) to 3 (9.7%); CG: unchanged **•No. injured persons:** IG: 7 (22.6%) to three (9.7%); CG: two to three **•Falls occurrence**: pre: RR: 1.283 (95% CI: 0.384–4.289); post: RR: 1.529 (95% CI: 0.480–4.877)	**Av. Generalized Self-Efficacy Scale score:** •IG: pre: 69.1 +—7.0; post: 74.1 +—6.1 •CC: pre: 70.1 +—12.9; post: 67.4 + -9.9 **Av score on the Social Support Scale:** •IG: pre: 66.1 +—11.3; post: 69.8 +—12.0 •CC: pre: 70.0 +—9.3; post: 63.0 +—11.0
Jensen, 2002, Sweden [60]	CRCT, LTCF	•9 facilities; IG = 4, CG = 5 •Residents: aged 65 + , selected in a cross-sectional manner; (*n* = Baseline: IG = 208, CG = 194) (*n* = follow-up: IG = 167; CG = 157) •Staff: Permanent staff (273 nurses’ aides, 20 registered nurses & ext. employees, 8 PTs during int., 3 PTs at follow-up)	MFI	•Staff education •Environmental modification •Exercise •Supply or repair of aids •Change in medication •Hip protectors •post-fall problem-solving conferences	•Resident baseline assessment •All nurses were interviewed to determine the use of physical restraints and the number of falls (6 months prior)	•I: 11 wks •F: 34 wks	Usual care	•**No. residents with falls**: IG: 82/188; 44%; CG: 109/196; 56%; RR .78 (CI, 0.64–0.96); adjusted OR 0.49 (CI,0.37–0.65) •**No. falls:** IG: 273/40898 on observation day; CG:346/41590 observation day **•Incidence of falls (/1,000 person-days)**: IG: 6.7; CG: 8.3; adj. incidence rate ratio 0.60(CI, 0.50 to 0.73) **•Time to first fall:** adj hazard ratio 0.66 (CI,0.50 to 0.79)	
Kerse, 2004, New Zealand [61]	CRCT, LTCF	•Sites selected by dependency level: 8 high-level, 4 low-level, 2 high-level units with secure dementia units (IG = 7; CG = 7) •Residents: All older people with low or high-level dependency (*n* = Baseline: IG = 241; CG = 312; follow-up: IG = 177; CG = 239) •Staff: existing staff of each facility (*n* = N/R)	MFI	•Systematic, individualised fall-risk management using existing staff and resources •Using a fall-risk assessment tool •Using a high-risk logo for residents deemed to be at high risk of falling •Staff education (2–4 h)	•Resident demographic information •Dependency levels (composites for mobility, behaviour and self-care) •Falls and fall-related injuries (3–5 months prior)	I and F: 52 wks	Usual care	**•No. falls: IG: 173(56%), CG: 103 (43%)** (*p* < .018) during the int. period •**Incidence rate of falls**: IG > CG (adj. IRR = 1.34, 95% CI = 1.06–1.72) during int **•No. fall-related injuries**: 199 residents (26%) and 72% of fallers sustained injuries; 47 were serious injuries (IG = 34, CG = 20; 5 had > 1 serious injury) •**Fall- injuries rate** (adj. IRR 1.12.CI, 0.85–1.47) and fall-related serious injury rate (adj. IRR 1.14.CI, 0.61–2.13) *p* = NS	
Ray., 2005, US [62]	CRCT, LTCF	•112 facilities (IG = 56; CG = 56) •Residents: Aged 65 + , not bedbound; (IG: 4,932, CG:5,625) •Staff: fall teams appointed in each facility: nurse, 1–2 CANs, an OT, a PT and an engineer (*n* = N/R)	SI	Staff training (2 days) on 4 safety domains: living space and personal safety; wheelchairs, canes and walkers; psychotropic medication use; and transferring and ambulation	•Demographic info, mobility levels, falls history, psychotropic medication – from Mandatory Minimum Data Set (a year prior) •LTCF records to verify residence in the facility	I and F: 52 wks	Usual care	•**Fall-related injuries** (person-years)**:** 838/8,172 first injuries occurred; 270 were hip/femur fractures; 240 were other fractures; 328 were other injuries **• Occurrence injuries between groups** (/1,000 person-years): IG: 106 injuries; CG: 99.5 (Adj. RR, 0.98; 95% CI, 0.83–1.16)	
Meyer, 2009, Germany [63]	CRCT, NHs	•29 NHs per study group, with at least 30 residents, not using a fall risk assessment tool •Residents: aged 70 + , able to walk without assistive devices (*n* = 574 IG; *n* = 551 CG) •Staff: nursing staff attending the educational session	SI	•Education session (60–90 min) on fall risk assessment and using the Downton Index	•Baseline characteristics of clusters and resident participants •Cluster adjustment of data avoided to present the raw baseline characteristics of the study population	I and F: 52 wks	Education without Down Index tool	**•No. residents with > 1 fall**: IG: 52%; CG: 53%, *p* = .88 •**No. falls**: IG: 1,016; CG: 1,014 •**Mean incidence of first fall (/month**): IG: 0.084 ± 0.046; CG: 0.082 ± 0.042 (*P* = 0.85) •**Mean incidences, all falls (/month)**: IG: 0.162 ± 0.108; CG 0.167 ± 0.084 (*P* = 0.57)	
Colon-Emeric., ^B^2017, US [64, 73–75]	CRCT, NHs	•24 NHs, IG = 12, CG = 12 •Staff: All full-time staff able to understand English (*n* = IC: 658; CC: 743) •Residents: aged 65 + with > 1 fall in either data collection window and remained in facility for > 30 days after (*n* = IG: 887; CG: 907)	MCI	•CONNECT programme: 3 main components, delivered over 3 months: CONNECT and learn protocol; relationship mapping; unit-based mentoring protocols •Followed by a FALLs programme delivered over 3 months: training session and team teleconference meetings, staff education, post-fall problem solving, audit & feedback	•Staff surveys (openness, communication, accuracy, timeliness); Participation in Decision-making Instrument; Safety Organizing Scale; Local Interaction Scale; Perceived Quality of Care Scale (2 wks. prior) •Random 50 residents/site: medical records (6 mths prior)	•I: 39 wks •F: None	FALLs programme only	**O measurements**: 3 months after CONNECT; 3 months after FALL; (3 months after completing interventions) •**Recurrent fall rate**: IG: 4.06, IQR, 2.03–8.11; CG: 4.06, IQR, 2.03–8.11 •**Injurious fall rates (/resident/year**): IG: IQR, 0–2.21, Mean 2.07, SD (4.56); CC: 0–2.12, Mean 2.07; SD (4.56) (no diff) •**Fall risk reduction activities:** IG: Mean 3.3, SD 1.6; CG: Mean 3.2, SD 1.5	**Staff surveys (openness, communication, accuracy, timeliness); Participation in Decision-making Instrument; Safety Organizing Scale; Local Interaction Scale; Perceived Quality of Care Scale:** completed by 1,545 unique staff members, including IG:734 (37%); CC: 811 (44%); the findings were significant only for the improvement in staff communication timeliness measure: 0.8 [SE,0.03] points on a 5-point scale; *p* = .02
Ward, 2010, Australia [65]	CRCT, LLTCF	•88 aged care facilities; *n* = IG: 46; CG: 42, 6 of whom withdrew •Residents: older, mixed patient type (high-care, low-care and dementia-specific) (*n* = 5391) •Staff: all staff (*n* = N/R)	SI	•Employment of project nurse to encourage staff in fall prevention interventions (risk assessment, mobility assessment, hip protectors, calcium and vit. D supplements, continence management, exercise programmes, appropriate footwear, medication review, post-fall management review	•The number of falls, fall-related injuries (fracture, hospitalisation and death) (7 months prior)	•I: 74 wks •F: None	•Usual care	**•Vit D (/100 beds):** pre: IG: mean 6.7 (95% CI, 1.2 to 10.9); CG mean 12.7 supplements (95% CI, 7.4 to 18.1); post: mean 2.0 supplements (*P* < 0.001) •**Hip protectors **(/100 beds)**:** pre: 5.1 both groups (95% CI, 3.1–7.0); post: both increased: first stage slope, 0.25 (95% CI, 0.06–0.43; *P* = 0.008); second stage slope, 0.29 (95% CI, 0.17–0.41; *P* < 0.001) **•Falls rate (/100 beds/month):** no change either group 16.0 (95% CI, 14.2–17.9); pre: (0.14 falls; 95% CI, − 0.17–0.45; *p* = 0.37); post: (− 0.023 falls; 95% CI, − 0.14- 0.09; *p* = 0.686) •**No. femoral fractures:** CG: 106; IG: 109 •**Rates femoral neck fractures:** similar in both groups (*p* = 0.8) •Overall, no difference in groups regarding no. of falls & related injuries	
Bouwen, 2008, Belgium [66]	CRCT, NHs	•10 nursing wards from 7 NHs; *n* = CG: 5 wards; IG: 5 wards •Residents: all ‘conscious’ residents; *n* = IG: 210; CC: 169 •Staff: all staff nurses (*n* = N/R)	MCI	•Staff training on risk factors for falls •Environmental modification •Use of fall diaries	•Questionnaire: fall risk factors, chronic medication and co-morbidity (IG only) •Baseline assess: mobility (TUGT), cognition (MMSE) •Accidental falls: (6 months prior); both groups	•I: 6 wks •F: 26 wks	•Usual care	•**No. falls (at least once):** pre: IG: 44/210 (21%). CG: 20/169 (12%); post: IG: 28/203 (14%); CG: 38/158 (24%) •Difference between av. no. falls in the two groups: (*p* = 0.10); (OR: 0.46 (95% CI = 0.26–0.79)	
Lomax, 2020, US [67]	QI, pre-post-test, SNF	•164-bed single-site: skilled nursing, rehabilitation and respite care services (75% in LTCF) •Residents: aged 65 + in LTCF part •Staff: convenience sample: RNs and CNAs (*n* = N/R)	SI	•Staff education to implement post-fall huddles	•Demographics and characteristics of residents; fall data at baseline	•I: 8 wks •F: None	N/A	**•No. falls:** pre: 68/164 (75.6%); post: 22/164 (24.4%) •**Timing of fall**: night shifts (pre: *n* = 43,47.8%; post: *n* = 31,45.6%; *p* < .732) •**Precipitants:** walking, leaving beds, using wheelchairs •**No. staff on unit:** pre: 1–2 members (*n* = 31, 45.6%), 2–3 (*n* = 20, 29.4%), 3–4 (*n* = 10, 14.7%), 4–5 (*n* = 7, 10.3%); post: 1–2 (*n* = 12; 54.5%), 2–3 (*n* = 7, 31.8%), 34 (*n* = 2, 9.1%), 4–5 (*n* = 1, 4.6%); *p* < .724	
Wells, 2011, US[71]	Pre-post-test, SNF	•3 units (single-site facility) •Residents: required total care or rehab services (*n* = 180) •Staff: CNAs (*n* = 90); had to complete pre-post-test and attend educ. (*n* = 42) to be included in analysis	SI	• Staff education	•Staff knowledge and attitude survey developed by investigators •No. falls (3 months prior)	• I: 13 wks •F: 13 wks	N/A	**O**: at 3 months and 6 months **•No. falls:** pre: 109 fallers; post: 86 fallers, F: 52 fallers **•Fall rate:** reduced (52%)	**Staff knowledge test:** The pre-post score was improved, *p* = .322
Ofosuhen, 2021, US [68]	QI (pre-post-test), LTCF	•Single-site: nursing, non-nursing and residential care •Staff: all RNs & LPNs (*n* = 55) •Resident: aged 65 + (*n* = 120)	SI	•Staff education using the STEADI Fall Risk Assessment Toolkit	•Staff nurse knowledge using survey regarding STEADI (a week prior) •Falls rate (3 months prior)	• I: 4 wks • F: None	N/A	**•Fall rate (%):** pre: 59.8%; post: 23.3% **•Av. no. fallers (/month):** pre: 19 fallers; post: 7 fallers	**Staff knowledge test:** pre-test: 12 out of 55 (21.8%) had correct responses to all 13 questions; post-test: 96.3%; Pre-post-test comparison: 75% increase in staff knowledge
Hurst, 2019, US [72]	Capstone project (pre-post-test), LTCF	•Single-site (169 beds) •Residents: aged 65 + , need assistance ADL or skilled care, > 1 prior fall (*n* = 154) •Staff participants: all staff (*n*: N/R)	MCI	•Staff education to implement the Morse Fall Scale Assessment Tool, identifying fall risks and developing a care plan •Falls champion	•The fall rate (3 months prior) • Resident demographic data	•I: 13 wks F: None	N/A	**•The fall rate:** pre-intervention: 59 falls (10.4%) post-intervention: 29 falls (5.1%)	
Ogundu, 2016, US [69]	Pre-post-test, SNF	•Single-site; 100-bed, for-profit •Staff: full-time, part-time or per diem nursing staff, trained on the hourly rounding and bed alarm intervention (*n* = 40) •Residents: N/R	MCI	•Staff education •Implementation of hourly rounds •The use of bed alarms	•No. falls (5 months prior)	• I: 26 wks •F: None	N/A	•**No. falls:** pre: post: **•Fall rate by shift:** night shift: pre: post: day shift: pre: post: evening shift: pre: post: **•**%, residents with any fall: pre: 36.1%; post-: 22.8%	
Aguwa, 2019, US [70]	Pre-post-test, LTCF	•Single-site •Staff: all nursing & nursing assistant staff (28/46 members: 7 nurses, 21 nursing assistants) •Residents: aged 65 + (*n* = 114)	SI	•Staff education using the AMDA fall prevention guideline and the STEADI Fall Risk Assessment Toolkit	•Staff self-efficacy using SEPF-N and SEPF-A scales	•I: 26 wks •F: None	N/A	N/A	**Staff self-efficacy:** Pre-test: 25% nurses didn’t give direct report to NA about residents’ fall risk, or info. on how to prevent falls. 40% of NAs said they didn't receive a verbal report about residents’ fall risk; 90% didn’t receive info. on fall prevention Post-test: All nurses said they would include fall prevention strategies into their communication and shift changes. All nurses agreed to improve teamwork. Self-confidence of nurses and NAs improved (90% & 100%, respectively)

*US* United States, *LTCF* Long-term care facilities, *NH* Nursing home, *SNF* Skilled nursing facility, *QI* Quality improvement project, *CRCT* Cluster randomised controlled trials, *SVH* State Veterans Home, *CG* Control group, *IG* Intervention group, *RN* Registered nurse, *LPN* Licensed practical nurse, *CNA* Certified nursing assistant, *OT* Occupational therapist, *PT* Physical therapist, *SI* Single intervention, *MCI* Multicomponent intervention, *MFI* Multifactorial intervention. *FMP* Falls management prevention, *I* Intervention, *F* Follow-up, *RR* Rate ratio, *OR* Odds ratio, *CI* Confidence interval, *IQR* Interquartile range, *SD* Standard deviation, *IRR* Incidence rate ratio, *O* Outcome, *N/A* Not applicable, *N/R* Not reported, *Adj*. Adjusted, *No*. Number of, *Av*. Average

### Risk of bias in studies

The quality appraisal scores for the seven cluster RCTs ranged from six to nine out of the 13 criteria in the JBI tools. Three studies scored 9 s, while three studies scored 8 s, and one study was assessed as having six of the evaluation criteria. The lack of the blinding of participants and of those who administered the interventions was the weakest area of the cluster RCTs included. The 20 quasi-experimental study scores ranged from four to seven for the nine criteria of the JBI checklist; 10 studies received 5 s, while seven studies received 6 s, two studies received 4 s and one study scored 7 out of all the appraisal criteria, as the use of control groups was limited in the studies included. As planned, no studies were excluded based on the quality appraisal results; all studies included were of low to moderate quality (Additional file 2).

#### Implementation strategy descriptions

Across 27 studies, this review identified 39 implementation strategies used in falls prevention interventions, of which 36 strategies were aligned directly with the ERIC list. Three strategies from the ERIC Taxonomy were added to better reflect the implementation approach: once-off training, dynamic education and the provision of ongoing medical consultation strategies. The number of discrete implementation strategies per study varied from 3 to 17. Table 3 provides an overview of the ERIC implementation strategies used, grouped into nine ERIC subheadings, and “Additional file 3” provides detailed descriptions of the implementation strategies in all studies.

**Table 3:**
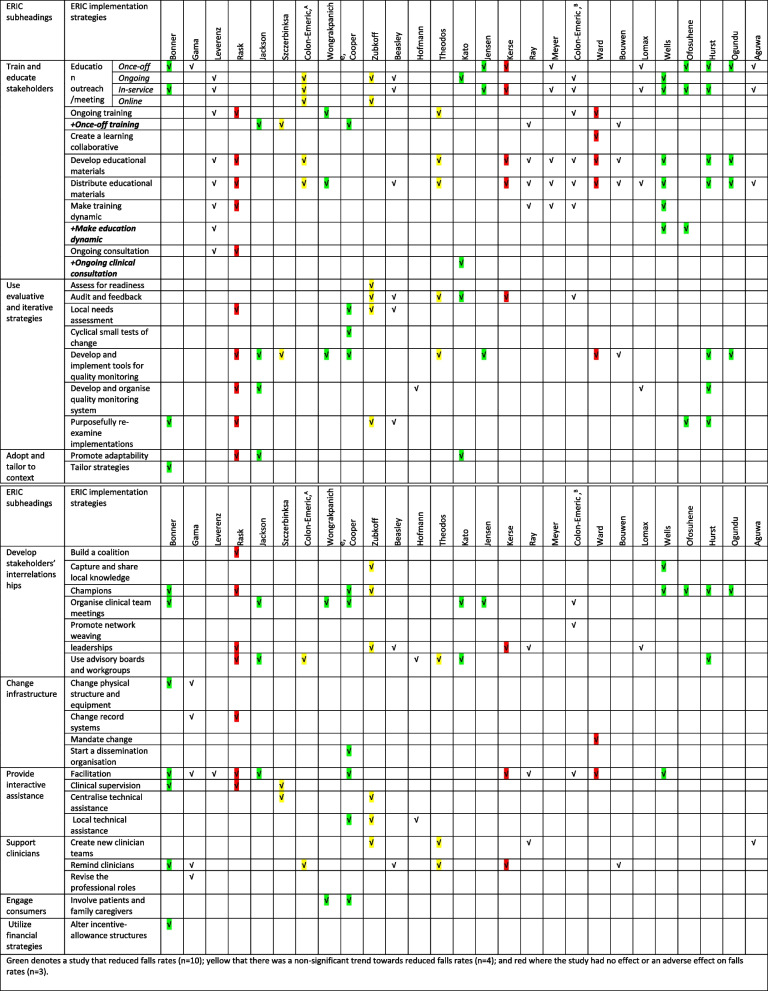
Implementation strategies used in LTCF falls preventions interventions

Green denotes a study that reduced falls rates (*n* = 10); yellow that there was a non-significant trend towards reduced falls rates (*n* = 4); and red where the study had no effect or an adverse effect on falls rates (*n* = 3)

Most studies used multiple implementation strategies to support the delivery of the fall prevention intervention. As per Table 3, one study reported implementation strategies in eight categories [46], two studies in six categories [49, 54], three studies in five categories [50, 55, 61], while eight [47, 53, 56–58, 62, 64, 65] and ten [51, 52, 59, 60, 66–69, 71, 72] respectively reported implementation strategies in either four or three categories. Three studies [48, 63, 70] discussed implementation strategies in two and one categories, respectively.

The most frequent categories of implementation strategy in the studies included in this review related to the following: training and educating stakeholders (*n* = 26); the use of evaluative and iterative strategies and the development of stakeholders’ interrelationships (*n* = 20); providing interactive assistance (*n* = 14); supporting clinicians (*n* = 10); changing infrastructure (*n* = 5); tailoring to the context (*n* = 4); engaging consumers (*n* = 2); and utilising financial strategies (*n* = 1). The results are organised according to these groups.

#### Train and educate stakeholders

The three most common implementation strategies used in this group were conducting an *education outreach/meeting* (*n* = 17), *distributing educational materials* (*n* = 17) and *developing educational materials* (*n* = 13) (for more details, see “Additional file 3”). In the 17 studies which involved an education outreach/meeting for improving staff knowledge, 12 studies of which [46, 48, 56, 60, 61, 63, 64, 67, 68, 70–72] involved in-service education sessions at organisational sites; one study [55] included a virtual education session, and one study incorporated in-person and teleconference sessions [52]. Ten studies included once-off education sessions ranging from a number of hours in a single day to a number of days in a single week [46, 47, 60, 61, 63, 67–70, 72]. Seven studies involved ongoing education sessions, such as monthly or quarterly [48, 52, 55, 56, 58, 64, 71].

In total, 17 studies distributed educational material physically or online (e.g., via notebooks, posters, brochures or manual training materials [48, 49, 52, 53, 56, 57, 61–67, 69–72]. Thirteen studies involved the development of new educational materials related to interventions (e.g., presentations, falls forms, videos, modules) [48, 49, 52, 61–66, 69, 71, 72, 77]. Six studies focused on improving staff skills via frequent ongoing training (i.e., workshops) [48, 49, 53, 64, 65, 77],whilst five involved once-off training days/hours, also in the form of workshops [50, 51, 54, 62, 66]. We identified two studies that concerned both staff education (knowledge) and training (skills) [48, 64]. The strategy of making training dynamic was used in five studies by varying the learning style of training to ensure it was interactive (e.g., group discussions, storytelling and role play, problem-solving) [49, 62–64]. One study used dynamic education strategies (i.e., acronyms, pictures, videos) [68], and two studies used dynamic strategies for both training and education [48, 71]. Two studies [48, 49] provided ongoing consultation to support the implementation strategy needed for fall prevention, whereas one study involved ongoing clinical consultation with experts to solve medical problems [58]. One study fostered a collaborative learning environment by creating a specific collaborative learning strategy connecting all staff participating in the intervention with the fall prevention resources and encouraging them to use them through continuous networking meetings [65].

#### Developing stakeholder interrelationships

Eight studies used the *identification and preparation of champions* for supporting implementation in sites, [46, 49, 54, 55, 67–69, 72], while one study only reported the process of identifying champions (ref single study). Seven studies used *advisory workgroups* including formal groups of falls teams or multi-disciplinary committees to provide recommendations on implementation [49, 50, 52, 58, 59, 72, 77]. Seven studies *held weekly/monthly meetings* with clinician implementation teams to reflect implementation efforts and issues as an integrated part of interventions [46, 50, 53, 54, 58, 60, 64]. Six studies employed *leadership recruitment, design and training* to support changes in care plans, monitor staff performance and offer intervention recommendations [49, 55, 56, 61, 62, 67]. Two studies *captured and shared local knowledge* of staff to allow them to discuss their experiences with current fall issues and techniques they applied to prevents falls [55, 71]. One study *promoted staff to weave a network* by creating a group-to-group and individual-to-individual relationship map to improve staff interaction and communication [64]. One study used *a coalition-building strategy* by describing all of the interventions and activities that were required to participate in the study [49].

#### Using evaluative and iterative strategies

Eleven out of the 27 studies *developed and implemented tools for quality monitoring* (i.e., falls forms, flow charts, log, checklists, staff diaries) [49–51, 53, 54, 60, 65, 66, 69, 72, 77]. Six studies *purposefully re-examined the implementation* to assess the success of, and impediments to, interventions and to track progress [46, 49, 55, 56, 68, 72]. Six studies c*onducted audits and provided feedback* by informing staff about the implementation outcomes and progress [55, 56, 64, 77]*,* and two of them only conducted audits, without feedback [58, 61]. Five studies *developed and organised quality monitoring systems* for monitoring the outcomes using software programs, or tracking reports, or via clinical documentation processes [49, 50, 59, 67, 72]. Four studies *conducted local needs assessments* relevant to their current fall approaches to determine the need for interventions [49, 54–56]. One study *assessed readiness* [55], and one included five *small cyclical test* changes to complete a fall risk intervention tool with refinement in each cycle [54].

#### Providing interactive assistance

Eleven studies employed *facilitation strategies* that provided staff encouragement and support in their responsibilities as implementers of the intervention, in problem-solving processes and in managing interpersonal staff communication problems, using researchers, organisational coaches, external consultants and paid facilitators [46–50, 54, 61, 62, 64, 65, 71]. Three studies used local professionals’ (e.g., nurses, coaches) to *provide technical assistance* for implementation concerns [54, 55, 59]. Three studies involved *clinician supervision* (e.g., weekly supervision visits) [46, 49, 51]. Two studies included *centralised technical assistance* systems such as weekly visits to support staff for the purpose of filling in falls forms and open discussion calls to answer questions and troubleshoot [59, 63].

#### Supporting clinicians

Clinicians were given *reminders* in seven studies via electronic reminders in the registration systems or labelling/colouring dots on residents’ profiles, on armbands, or reminders in residents’ rooms [46, 47, 52, 56, 61, 66, 77]. Four studies *created new clinician teams* to implement interventions [55, 62, 70, 77]. One study *revised professional roles* by outlining their responsibilities [47].

#### Changing infrastructure

Two studies *changed the physical structure and equipment* in sites (the placement of laundry receptacles in hallways, the use and storage of housekeeping and maintenance equipment, and the location of a fall registry) [46, 47]. Two *changed record systems* on falls risk assessment tools in the registration systems [47, 49]. One study *mandated change* by obtaining support for the interventions from divisions of general practice [65]. One study started to *disseminate information* in a more organised way, as two senior care assistants and two nurses on site took responsibility for initiating huddles [54].

#### Adopting and tailoring to the context

Three studies *promoted adaptability of the intervention* (e.g., exercise interventions or care plans tailored to the residents' needs based on assessment and staff communication procedures) [49, 50, 58]. One study used *tailored strategies* to address barriers (e.g., translating educational sessions for non-English-speaking staff) [46].

#### Engaging consumers

Two studies involved family caregivers in educational sessions and root cause analysis activities [53, 54].

#### Utilising financial strategies

One study *altered incentive/allowance structures* by compensating staff who attended educational sessions on their days off [46].

#### Implementation outcomes description

Implementation outcomes were identified and synthesised from 15 of the 27 studies, with four studies reporting two or three implementation outcomes [52, 53, 55, 57]. Table 4 shows how each study's implementation outcomes were measured. Most studies used both administrative data (i.e., medical records or other documentation) and/or self-reported data (audits of staff/facilities, self-reports) to assess implementation outcomes. *Fidelity* (*n* = 8) was the most commonly reported implementation result outcome [47, 49, 50, 52, 55, 56, 61, 62], measured as the level of compliance with interventions conducted, at either the facility level or the staff level. Three studies evaluated the *acceptability* of the training by surveying staff satisfaction [48, 53, 55], whereas one study reported *acceptability*, but this was at the level of the resident*,* based on how many of them did not agree to do their recommend exercises or activities, which was documented by monitoring staff [57]. Similarly, three studies assessed adoption by determining the percentage of residents who completed entire fall risk assessment and exercise regimens from all residents referred to a physiotherapy programme and could be evaluated, as well as the uptake of interventions by staff at each facility [51, 55, 64]. One study measured the level of participation in executing the programme at the facility, to determine *penetration* [52]. One study evaluated *feasibility* by monitoring the implementation process for completing fall intervention tools over The Plan-Do-Study-Act (PDSA) cycles; the falls champions were unable to complete the use of the tools when tested among new residents [54]. Only one study reported *appropriateness,* but this was at the level of the resident, based on residents' rejection of an exercise programme due to their health status, and their performance was monitored by staff [77]. One study reported the nursing care *implementation cost* by calculating how long it takes an average nurse to complete a falls assessment risk tool (Downton index) and multiplying it by the nursing wage per hour [63]. The sole implementation outcome that was not addressed in any of the studies presented was *sustainability*.

**Table 4:** implementation outcomes of included studies

First author,	Implement. outcomes (Proctor 2011) [34]	Assessment	Sample size (n)	Outcome measurement and methods	Level of analysis	Results
Bonner	Not reported					
Gama	**Fidelity**	Pre-post evaluation of quality criteria compliance,	Facility: single-site Residents:127	Compliance with process criteria related to fall risk assessment measurement: twice (before/after interventions): cross-sectional random samples of medical records from the 127 residents who could walk 5 m independently	Staff	**Pre-intervention:** generally poor; the most problematic criteria concerned “orthostatic hypotension”, “review of assistive devices” and “strength and balance”, whereas only the “recording fall characteristics” criterion achieved compliance. **Post-intervention**: all criteria were improved except the eyesight evaluation criterion
Leverenz	**Acceptability**	Survey	Facility: single-site Staff: 8	Staff satisfaction with the manner of collaborative training: A post-training survey	Staff	75% were strongly satisfied with group training, less satisfied with individual training sessions, and 63% provided neutral responses to the survey item. 75% of staff strongly agreed that their active participation feedback was welcomed and encouraged
Rask	**Fidelity**	Pre-post-audit of the care process	Facility: 14 NHS medical records of 137 residents	Care process documentation: audits of records concerning 10 residents who had fallen twice or more before/during the 6-month evaluation period in both IG and CC	Facilities	Except for two, all critical areas of documentation concerning the assessment and management of fall risk factors improved. Notable advancements likely to directly impact recurrent falls included better assessment of fall risk factors, interventions to reduce the risk of falling and the likelihood of injury, and correction of environmental and equipment hazards
Jackson	**Fidelity**	Self-reported staff round sheets checklist	Facility: single-site Staff:10, documenting on 144 sheets a total of 454 residents	Average compliance regarding staff documentation of hourly rounding: Staff frequency and consistency noted in each unit for every two-hour rounding sheet, with a focus on the 4 Ps (potty, pain, positioning and possession)	Staff	2H patient rounding: average compliance was 91% across the three nursing units
Szczerbinksa	**Adoption**	Quantitative measurement of intervention implementation level	Facility: 3NHs Residents: TUG test: (N: A:80, B:70, C:36) POMA test: (N: A:48, B:54, C:27) Residents who were referred to a rehab programme: (N: A:9, B:25, C:22) Residents who completed a rehab programme (N: A:9, B:23, C:20)	Measurement of the application rate of two fall prevention tests (TUG test and POMA test) and rehab programme at 3 sites: ratio of residents assessed via the two tests to all residents able to be evaluated in all NHs; ratio of residents at risk of falling and participating in a rehab programme to all residents referred by physicians to the programme	Facilities	•The implementation rate of risk assessment at the first stage (using a TUG test): mean: NHA 87.9%, NHB: 86.4%, NHC: 97.3% •The implementation rate at the second stage (using a POMA test): mean: NHA:88.9%, NHB: 100%, NHC: 100% •The implementation rate at the third stage (rehabilitation programme): mean: NHA:22.5%, NHB: 75.7%, NHC: 100%
				The percentage of residents who completed the physiotherapy programme from all residents referred to it	Residents	The percentage of residents who completed the physiotherapy programme was over 90% in all NHs. Among all residents in NHs, only 9.1% in NH A, 13.3% in NH B and 38.6% in NH C were involved in the rehabilitation intervention
Colon-Emeric, ^A^	**Fidelity**	Pre-post facility (self-reported)	Facility: 36 NHs	The average compliance rate of fall risk assessment: facility self-reported data with fall prevention change concepts (fall risk assessment, labelling, post-fall risk assessment, risk factor reduction)	Facilities	**Self-reported compliance:** •The assessment of the fall risk level increased from 86 to 100% •Labelling of high-risk residents increased from 75 to 99% •The proportion of high-risk residents reported as having multiple-risk-factor reduction completed increased from 62 to 99% •post-fall assessments improved, rising from 81 to 98%
	**Penetration**			The level of facility participation crosses collaborative activities: facility self-reported data in how consistently submitted data reflecting the level of participation to the fall prevention change concepts		**Facilities were classified on their level of participation across all activities** •High: *n* = 5, participated in 66% or more of activities •Medium: *n* = 16, participated in 33% to 66% of activities •Low: *n* = 15, participated in less than 33% of activities
	**Fidelity**	Pre-post chart abstraction documentation	Documented from 1,398 residents’ records	Compliance on documentation of the process measures of the fall prevention change concepts: chart abstraction of 832 medical records		**Chart abstraction compliance**: modest improvements in screening of falls (51% to 68%, *p* < .05), risk-factor reduction (4% to 7%, *p* = .30) and medication assessment (2% to 6%, *p* = .34); vitamin D prescription increased (40% to 48%, *p* = .03), and sedative-hypnotic use decreased (19% to 12%, *p* = .04)
Wongrakpanich	**Acceptability**	Survey	Facility: single-site Staff: 12 Residents: 32	Staff Satisfaction with training: A post-training survey	Staff	11/12 (91.7%) staff strongly agreed: training sessions for checklist implementation and fall prevention knowledge were useful. All participants agreed (75%) or strongly agreed (25%) that the checklist does not interfere with routine patient care time. 7/12 and 5/12 were very satisfied and satisfied, respectively, with the STOP-FALLING project
	**Adoption**	The most/least common intervention used from checklist		Quantifying the most/least frequently used intervention across all staff		Teaching (patient and family education), medication review, and in-house safety evaluation were the 3 most common interventions implemented by staff (32/32 patient checklists, or 100%). Low bed application was the least common intervention implemented (2 of 32; 6.3%)
Cooper	**Feasibility**	Process of intervention tool completion	Facility: single-site Residents: newly admitted (*n* = 29)	Process measure for completing fall intervention tools: In PDSA cycle 2, falls champions tested the tool as a group with new residents, who were unable to complete it	Staff	Process measures: 3 tools not completed, 6 fully completed (median duration: 22 days). Intervention tool (handover of actions) identified difficulties achieving process reliability while care assistants completed the tool. This prompted the invitation of a nurse into the improvement group and subsequent tool testing within the admission & care planning processes. 33.33% residents had completed risk/intervention tools
	Not clear				Staff	Balancing measure: The impact the project had on staff was explored. The responses were compiled into a Wordle, and the most frequently reported words included the following: "falls", "communication", "information" and "huddles"
Zubkoff	**Acceptability**	Survey	Facility: 26 SVHs Staff: 26 fall teams	Staff Satisfaction with educational call training: A post-training survey	Facilities	**Staff satisfaction**: 60 (69%) attendees described the calls as "Very or Extremely informative". 56 (66%) rated the calls as "Very or Extremely engaging". 68 (81%) would recommend the calls to a colleague
	**Fidelity**	Audit of report submission		Av. monthly report submission by teams: Self-reported document on delivery interventions	Facilities	**Report submission**: 19 out of 26 teams (73%), ranging from 65 to 85% of submissions each month
	**Adoption**	The most common intervention used out of 27 interventions		Quantifying the most frequently used intervention across all teams: Self-reported document on the delivery of 27 interventions	Facilities	27 interventions; most commonly implemented interventions: post-fall huddles (*n* = 19 teams), staff education (*n* = 15 teams), intentional rounding (*n* = 13 teams) and programme evaluation interventions (meeting to review fall cases or review post-fall huddle data) (*n* = 12 teams). However, differentiating staff assignments, the intervention was implemented by 6 teams and not covered by the project
Beasley	**Fidelity**	Pre-post audit of fall incidence tracking, and staff attending education	Facility: single-site Staff: 20 Residents: 20	Av. attendance of staff members at falls education and training in the previous 12 months: audit of training records and questioning of staff regarding their attendance	Staff	The level of compliance • the education programs are available to health-care workers: pre-implementation: 2/20 (10%)/ post-implementation: 19/20 (95%) (*P*-value = 0.0038)
				Av. compliance for tracking the incidence and prevalence of falls in uniform resident management systems: audit of chart residents and incident forms	Staff	The level of compliance •Tracking the incidence and prevalence of falls in uniform resident management systems: pre- implementation: 19/20 (95%)/post-implementation: 20/20 (100%) (*P*-value = 0.0038)
Hofmann	Not reported					
Theodos	**Acceptability** **Appropriateness**	Audit of resident’s non-completion of exercise program	Facility: single-site Documentation of 145 residents	Av. number who did not complete the fall exercise programme: audit of residents’ documentation of why they did not complete their recommended programmes, based on duration, frequency and level of activities. The therapist reviewed the documentation regarding the completion of the exercises	Residents	Not all residents were appropriate or agreeable to any form of exercise or activity. 8% of the residents refused to do any exercises; some were too ill, in pain, combative, experiencing contracture or in hospice services. The restorative or activities staff members were responsible for monitoring the residents’ performance of exercise interventions
Kato	Not reported					
Jensen	Not reported					
Kerse	**Fidelity**	Chart audit of fall reported forms	Facility: 7 Residents:123	Av. completion of fall risk assessment and implementation of the recommended intervention among residents: chart audit of fall-reporting forms	Facilities	Programme compliance rates: 5/7 IG had high compliance rates, assessing 48% to 85% of residents (noncompliant homes, assessed 0% and 35% of residents). On average, 49% of residents underwent individualised assessment (68% excluding the two non-compliant homes). After 5–7 months: 98% compliance, including the two non-compliant homes; 78% of all residents involved in the programme had fall-prevention strategies applied
Ray	**Fidelity**	Pre-post administrative data abstraction	Facility: IG = 56 Residents: 4,932	Av. facility compliance for implementing the safety programme (assessment and intervention) among residents at high risk: administrative data of mandatory minimum data set or facility records and nursing home records	Facilities	Facilities’ compliance with the programme: 52% of residents in intervention facilities complied with less than two thirds of the study programme recommendations (26% complied with less than 33% of recommendations; 26% complied with between 34% and 66; 48% complied with more than 66%)
Meyer	**Cost**	Nursing care implementation cost of using the assessment tools	Facility: 11	Economic evaluation: nursing staff of 11 nursing homes in the IG were asked how long it took on average to fill in the Downton Index	Facilities	Based on nurses' responses, there was an average of 3 min of nursing staff time per Downton Index per resident (n = 574 over an average follow-up of 10.8 months), resulting in a total of 310 h of nursing time. Fifteen minutes of nursing staff time per resident for the initial application of the Downton Index, including a full assessment of medication data, sensory deficits and mental state, were added, which means a further 143.5 h of nursing time. Overall, we estimated that the Downton Index required 475.25 h of nursing time. The calculation was based on the nurses’ gross salary of 22 euro per hour in 2006, as indicated by the finance department of a nursing home in Hamburg. Thus, using the Downton Index in this study yielded an approximate total of €10,500 ($16,170, £8,160), which was spent without a measurable clinical benefit
Colon-Emeric,^B^	**Adoption**	Quantitative measurement of intervention components received	Facility: 12 Staff: 658	Average intervention level received: the number of CONNECT intervention components each staff member had completed (0–10)	Facilities	Intervention dose varied across facilities (mean number of intervention components received by staff ranged 2.3–4.2 out of 10 possible
Ward	Not reported					
Bouwen	Not reported					
Lomax	Not reported					
Wells	Not reported					
Ofosuhene,	Not reported					
Hurst	Not reported					
Ogundu	Not reported					
Aguwa	Not reported					

Implementation Outcome Framework (Proctor et al., 2011) [34]: Acceptability: Perception among implementation stakeholders that a given treatment, service, practice or innovation is agreeable, palatable or satisfactory; Adoption: Intention, initial decision or action to try or employ an innovation or evidence-based practice; Appropriateness: Perceived fit, relevance or compatibility of the innovation or evidence-based practice setting, provider or consumer and/or the perceived fit of an innovation to address a particular issue or problem; Penetration: Integration of a practice within a service setting and its subsystems; number of eligible persons who use a service, divided by the total number of persons eligible for the service; number of providers who deliver a given service or treatment, divided by the total number of providers trained in or expected to deliver the service; Feasibility: Extent to which a new treatment or innovation can be successfully used or carried out within a given agency or setting; Fidelity: Degree to which an intervention was implemented as prescribed in the original protocol or as intended by the programme developers; Cost: Financial impact of an implementation effort

#### Intervention effectiveness

Because the studies included varied significantly in terms of study aims, outcomes, assessments, intervention durations and follow-up timing, a descriptive summary of the interventions’ effects on primary outcomes was provided, such as fall-related outcomes and staff-related outcomes. Although the effectiveness of the interventions was not the primary focus of this SR, we described the interventions’ effects in Table 2, where the exact effect sizes and, confidence intervals and p values are presented where available. To present data on the types of strategies used in studies that reduced falls rates, and those that did not, we colour-coded Table 3 to show that non-significant or neutral/adverse studies for falls rates (*n* = 4; *n* = 3 respectively) used a similar range of implementation strategies to the 10 effective studies, highlighting to the absence of a clear pattern of which implementation strategies are most effective.

#### Fall-related outcomes

Overall, 25 out of 27 studies reported fall-related outcomes; fall rates and the number of falls were the most common clinical outcome reported in all studies, while a few reported fall-related injuries and fall-related circumstances. Regarding fall rates, 10 reported effective reductions of fall rates [46, 50, 53, 54, 58, 60, 68, 69, 71, 72], while four reported a reduction of falls without statistical significance [51, 52, 55, 77]. However, Rask et al. indicated that the fall rate in the intervention group remained unchanged [49]. Keres et al. reported that the fall rate increased and was higher in the intervention groups during implementation compared to control groups [61], while Ward et al. found no change in the fall rate for both pre- and post-intervention across both groups [65].

Twelve studies determined that there was a decrease in the number of falls [47, 53, 56, 58–60, 66–69, 71, 77]. In contrast, two study reported a reduction of falls without statistical significance between both groups [63, 66], and Keres et al. reported an increase in the number of falls in the intervention groups. Four studies reported on recurrent falls or reported the number of fallers. Wongrakapnich et al. indicated a reduction in frequent falls [53], whilst Kato et al. reported that the number of fallers increased in both groups [58]. Colon-Emeric et al.,^B^ reported no effect and no differences between groups concerning the recurrent fall rate [64]. Hofmann et al. reported the number of fallers and the percentage of recurrent falls during the follow-up year without making any comparisons or statistical analysis pre-post interventions [59].

Four studies reported on fall-related characteristics. Jackson et al. found that half of falls occurred during the day, and the majority fell in their bedroom. Hofmann et al. reported that the most common fall period was during the 3–11 p.m. shift, and Ogundu et al. reported that the evening shift had no reduction in the number of falls with their intervention. Meanwhile, Lomax et al. found that the night shift had a greater number of falls, and that rising from beds and walking were the most common activities associated with fall incidents.

Seven studies measured fall-related injuries; two of them reported a significant reduction of injuries from falls [53, 55], while one study indicated an increasing number of fall-related injuries in the intervention group [61]; Keto et al., conversely, reported that the number of injuries and the number of injured persons decreased significantly in the intervention groups compared to control groups, which had an increase in injured persons but no change in the number of injuries [58]. Two studies reported no statistically significant change in fall injuries in intervention or control groups [62, 64], while Jackson et al. reported the quality assurance documentation for the average number of injuries during the intervention [50]. Moreover, two studies reported fall-related fracture injuries; one indicated a reduction in hip fractures rate compared to before the intervention [59], while the other found no difference in the number of femoral fractures between both groups [65].

Six studies that were CRCTs reported no differences between intervention and control groups in the fall rate, fall-related injuries or recurrent falls [61–66]. In contrast, Jensen et al. indicated a statistically significantly reduced number of falls and fall rates between the intervention and control groups, with no differences in time to first falls between the two groups [60]. From the quasi-experimental studies, only three had a control group. Two reported no significant differentiation of fall rate between groups [52, 61], whereas another reported a relative reduction in the falls rate in the intervention group, although the number of fallers increased in both [58]. Some studies included other fall-relevant outcomes, (e.g., vitamin D prescription, etc.) which are detailed in Table 2.

#### Staff-related outcomes

Four studies measured staff knowledge using surveys; three reported a positive effect on improving their knowledge [46, 50, 68], whereas one reported no significant effect [71]. Three studies measured nursing staff self-efficacy and reported a positive impact [48, 58, 70]. Staff motivation and empowerment was measured in one study, and effective results were noted [58]. Staff communication was measured by Colon-Emeric et al.,^B^ with no effect nor difference between groups [64].

## Discussion

Many SRs have described intervention effectiveness as regards the clinical effectiveness of fall prevention interventions among LTCF residents; however, this is the first SR to synthesis the interventions’ implementation strategies and implementation outcomes in order to provide insight into how they have been used in LTCF fall prevention interventions. Two frameworks from the implementation science, namely the ERIC implementation strategy and Proctor implementation outcome taxonomies, were used to synthesise the implementation strategies used and outcomes, as these were described in various ways, with varying terms used for the same strategy in some cases.

The findings identified that 39 implementation strategies were used across 27 fall-prevention studies targeting LTCF staff. Training-and-education implementation strategies were the most popular. Other SRs also found that educational strategies were comprehensively used, targeting health workers to change their professional practice or behaviour [78–82]. A recent SR published in 2020, found that staff education interventions on how to prevent falls among residents have benefits for minimising falls and recurrent falls [22]. The studies included in this SR incorporated multi-faceted strategies and education sessions, and distributed educational materials were commonly used. The studies used varied frequencies and modes of education and training delivery. The delivery methods included formal lectures, teleconferences, education calls. Focussing on the frequency of education delivery (once or continuous) helped to understand the educational implementation process. We established subcategories regarding frequency and delivery mode related to education meetings/outreach strategies in order to clarify overlapping definitions. We also identified other non-ERIC-codable strategies related to staff education and training: once-off training, dynamic education, ongoing medical consultation.

Some studies focused on enhancing staff skills through hands-on training, whether once-off or ongoing, including different interactive learning and training activities (e.g., problem-solving skills). They showed a mixture of passive (e.g., distribute education material) and active (dynamic education/consultation) education strategies, as previous literature indicates that passive education strategies are highly likely to be ineffective for adherence [83]. However, not all studies reported the educational dose durations, which is an integral part of describing the education sessions. This is consistent with a previous scoping review, which found that there are many falls prevention education programmes available for health workers, but that many aspects of reporting education programmes are of poor quality [84].

Vlaeyen et al. identified 27 barriers for staff in terms of implementing fall interventions in LTCFs, reporting that a lack of staff knowledge and skills was one of the most common barriers, requiring a focus on fall prevention interventions, as it is considered a changeable factor [27]. This aligns with our review, where the most common strategies used for implementing fall prevention interventions in LTCFs were education and training, reflecting their perceived status as modifiable and influential on care. Moreover, identifying barriers and facilitators is a strategy that is considered a critical precursor to determining the best implementation methods and processes, allowing the use of evidence-based interventions to address the barriers. Additionally, it has been found that tailored strategies to address determinants of interventions during practice are considered effective for improving health worker performance, support for uptake, and determining an adequate implementation strategy [31]. This review identified limited use of evaluation of barriers/facilitators at the level of staff/facilities before conducting the interventions and hence tailoring of the implementation strategies to address barriers, with only one study doing so. This may have affected the success of the implementation of the others.

Our findings also identified that most studies used a combination of strategies for fall interventions (e.g., audits/feedback, facilitation, education outreach/meetings, clinicians’ reminders and leadership). These strategies have been identified as the most influential strategies in terms of success in complex interventions by supporting health workers in their real-world practice [29, 30, 32, 82, 85, 86]. Moreover, all of the studies used a varying number of strategies, ranging from three to 17 strategies. This review gained insight into strategies that were used regularly, as well as the multi-faceted nature of implementation strategies, in terms of the total number of strategies or the degree of use of strategies that are reported to be the most influential (e.g., audits/feedback), but it did not reveal the relative impact of single or combined implementation strategies. It is essential to consider the relationships between the quantity and combination of strategies used and the success of their implementation [87].

Although many studies measured implementation outcomes, 12 studies did not. Fidelity was the most commonly measured implementation outcome, in eight studies. It was reported based on levels of compliance with delivering the falls intervention(s), but this, when used as a sole implementation outcome, is not enough to assess behaviour change or full adoption of the intervention. The findings also revealed varying lengths of study duration and follow-up. Only a few studies included short-term follow-up measurements. Sustainability, one of Proctor et al.’s eight implementation outcomes, requires a follow-up period for the measurement of long-term compliance, and reflects the impact of intervention implementation in terms of continued acceptability, effectiveness and adaptability, as is required in a real clinical setting [88]; this review did not identify any study that reported on it.

According to the literature, the healthcare system is burdened by fall-related costs, reported to be approximately 4 million bed days (£2.3 billion per year), and 50% of these costs involve hospitalisation [8, 88]. However, we found only a few references to fall-related costs. One study employed one of the nine financial ERIC strategies, titled “Alter incentive/allowance structures”, to compensate staff who attended the education session on their day off, but otherwise there was an absence of the use of financial strategies, as found in other SRs [79]. Another considered the delivery cost as an outcome, without any cost–benefit analysis. More information related to fall-related costs is needed, as it is significant for implementing fall prevention interventions in LTCF. Indeed, a SR found that multifactorial fall prevention interventions were beneficial in reducing the fall rate in LTCF only when combined with external resources and financing [20].Economic evaluations are vital for clarifying the cost benefits of making clinical and policy decisions about fall prevention in LTCFs. This should be the focus of future implementation work in falls prevention interventions in LTCFs.

Although the majority of the studies in our SR reported a significant positive effect on fall-related outcomes, there was heterogeneity in the studies in terms of study aims, outcomes, assessments, intervention durations and follow-up timing, as previously reported by others [78]. Two of the studies measured staff-related outcomes (i.e., staff knowledge) only. In the others, there was variable reporting of falls indices such as fall rates, the number of fallers, and so on. This led us to narratively describe the results and, in this review, there was no discernible pattern of strategies used exclusively in effective fall prevention interventions. Future research on fall prevention must therefore explicitly describe the effectiveness of implementation strategies on implementation outcome and clinical outcomes.

Despite the fact that a wide variety of implementation strategies were identified, detailed reporting of how strategies were applied, along with the implementation outcomes in the studies included, were under-reported. Therefore, the effectiveness of fall interventions was often attributed to the programmes, without regard for what implementation strategies had the greatest impact. Ongoing efforts to operationalise and measure implementation outcomes is necessary, as has been previously described [79].

Thus, this review highlights that there is a lack of consistency in reporting implementation strategies and outcomes, leaving no possibility to conclude what eventually influences the prevention of falls among LTCF residents. This points to the need for more research to identify the relationship between the implementation strategies and both clinical and implementation outcomes in the future.

### Limitations and strengths

The present SR has made a novel contribution to implementation science by providing a comprehensive synthesis of implementation strategies used for LTCF fall prevention interventions. The SR’s processes and analyses were double-checked and reviewed. Even though we included only English and Arabic texts, we excluded only one paper based on language, so the potential impact of language bias is likely negligible. We included published theses to reduce publication bias; an extensive search strategy of multiple grey literature databases might have further reduced possible publication bias, but the quality of the data would likely be low. We did not exclude studies based on the quality appraisal scores, with a view to being as inclusive as possible of studies that reported implementation strategies and outcomes in LTCF fall prevention. The majority of the studies included were quasi-experimental studies. Others were cluster RCTs; however, this approach does not produce the same level of confidence as RCTs, and it makes it difficult to compare between studies. There is also a limitation to a narrative synthesis due to the heterogeneity of the studies included, as compared to meta-analysis.

The ERIC and Proctor implementation outcome taxonomies are seminal implementation frameworks that provide a unified language for clearly understanding implementation strategies and outcomes, respectively. However, ERIC definitions of strategies are broad, and they include many aspects that lead to overlap and conflict of some strategies’ definitions, which may hinder a judgement concerning the labelling of some strategies (e.g., conducting education outreach/meetings). Moreover, there was a wide variety in the degree of reported details regarding the components of strategies. The results were weighted similarly for all studies that used the same strategy. To completely comprehend the effects of strategies, more detailed description and standard reporting of the implementation strategies using precise terminology is required. Proctor et al. have provided recommendations on how to specify implementation strategies designed to improve specific implementation outcomes, including the following: naming, describing and specifying the strategies [28]. The use of specific implementation guidelines such as the Standard for Quality Improvement Reporting Excellence (SQUIRE) and the Standard for Reporting Implementation Studies (StaRI) could help to standardise descriptions of strategies [89].

## Conclusion

This is the first study to synthesis the comprehensive implementation strategies used in LTCFs as regards falls prevention interventions. Many implementation strategies have been used, with education being the most common. Outside of the ERIC lists, three novel educational strategies were identified: providing once-off training, dynamic education and ongoing medical consultation. This review highlighted difficulties in learning from the implementation of fall prevention interventions, especially in relation to poor reporting of the implementation strategies used and implementation outcomes, which should be improved and clearly defined in future studies. There was no discernible pattern of implementation strategies used in effective interventions; thus, future falls prevention research needs to clearly describe the implementation along with the clinical intervention, and both clinical and implementation outcomes need to be included.

## Supplementary Information

**Additional file 1. **Search strategy used and PubMed database search strategy results.

**Additional file 2: Table 1.** The quality appraisal results of included CRCTs (Experimental studies). **Table 2.** The quality appraisal results of included Quasi-experimental studies.

**Additional file 3.** Detailed description of implementation strategies identified.

**Additional file 4.** The codebook definitions of implementation strategies identified.

**Additional file 5.** Detailed description of the reasons for excluding papers.

## Data Availability

All Data generated and analysed during this review is available in the supplement file.

## Abbreviations

LTCFs: Long-term care facilities
WHO: The World Health Organisation
SR: Systematic review
MAs: Meta-analyses
RCT: Randomised controlled trial
PROSPERO: The International Prospective Register of Systematic Reviews
PRISMA-P: The Preferred Reporting Items for Systematic Reviews and Meta-Analysis Protocol
MeSH: Medical subject headings
ERIC: The Expert Recommendation for Implementing Change Taxonomy
JBI: The Joanna Briggs Institute
NICE: The National Institute for Clinical Excellence
SQUIRE: The Standard for Quality Improvement Reporting Excellence
StaRI: The Standard for Reporting Implementation Studies
