# Supplementary material for: Implementation strategies to support fall prevention interventions in long-term care facilities for older persons: a systematic review
Source: BMC Geriatr. 2023 Jan 25;23:47. doi: 10.1186/s12877-023-03738-z (PMC9878796; doi:10.1186/s12877-023-03738-z)
Supplement: Supplementary file 1 — Additional file 1. Search strategy used and PubMed database search strategy results. [file 12877_2023_3738_MOESM1_ESM.docx]

**Additional file 1: Search strategy used and PubMed database search strategy results**

| Search term strategy used in electronic databases | |
| --- | --- |
| 1- population and settings | “long-term care setting*” OR “nursing home*” OR “residential care setting*” OR “Residential facilit*” OR “institution care” OR “nursing care” OR “nursing facilit*” OR “continuous care” |
| 2- Intervention/ phenomena of interest | Faller* OR “accidental fall*” OR falling OR falls OR slip* OR “fall prevention” OR “falls prevention”  **AND**  Prevent* OR reduc* OR minimi* OR decreas* OR interven* |
| 3- outcome | Feasib* OR sustain* OR adopt* OR accept* OR appropriate* OR fidelity OR implement* OR uptake* OR adher* OR facilitat*OR barrier* OR accessib* OR penetrat* OR mechanism* OR mediat* OR driv* |
| Combination with Boolean operators | 1 AND 2 AND 3 |
| limitation | English and Arabic languages  Published since 2001 |

| PubMed database search strategy results | | | | | |
| --- | --- | --- | --- | --- | --- |
| **Search**  #8 | **Actions**  **…** | **Details** | **Query**  Search: **((#1) AND (#2)) AND (#3)** Filters: **Arabic, English, from 2001 -**  **2021** | **Results**  [617](https://pubmed-ncbi-nlm-nih-gov.ucc.idm.oclc.org/?term=%28%28%231%29%2BAND%2B%28%232%29%29%2BAND%2B%28%233%29&filter=lang.arabic&filter=lang.english&filter=years.2001-2021&timeline=expanded&size=100&sort=relevance) | **Time**  15:06:29 |
| #7 | **…** |  | Search: **((#1) AND (#2)) AND (#3)** Filters: **English, from 2001 - 2021** | [617](https://pubmed-ncbi-nlm-nih-gov.ucc.idm.oclc.org/?term=%28%28%231%29%2BAND%2B%28%232%29%29%2BAND%2B%28%233%29&filter=lang.english&filter=years.2001-2021&timeline=expanded&size=100&sort=relevance) | 15:06:21 |
| #6 | **…** |  | Search: **((#1) AND (#2)) AND (#3)** Filters: **from 2001 - 2021** | [658](https://pubmed-ncbi-nlm-nih-gov.ucc.idm.oclc.org/?term=%28%28%231%29%2BAND%2B%28%232%29%29%2BAND%2B%28%233%29&filter=years.2001-2021&timeline=expanded&size=100&sort=relevance) | 15:06:13 |
| #5 | **…** |  | Search: **((#1) AND (#2)) AND (#3)** Filters: **from 1978 - 2021** | [727](https://pubmed-ncbi-nlm-nih-gov.ucc.idm.oclc.org/?term=%28%28%231%29%2BAND%2B%28%232%29%29%2BAND%2B%28%233%29&filter=years.1978-2021&timeline=expanded&size=100&sort=relevance) | 15:06:05 |
| #4 | **…** |  | Search: **((#1) AND (#2)) AND (#3)** | [728](https://pubmed-ncbi-nlm-nih-gov.ucc.idm.oclc.org/?term=%28%28%231%29%2BAND%2B%28%232%29%29%2BAND%2B%28%233%29&sort&size=100) | 15:05:50 |
| #3 | **…** |  | Search: **Feasib* OR sustain* OR adopt* OR accept* OR appropriate* OR fidelity OR implement* OR uptake* OR adher* OR facilitat*OR barrier* OR accessib* OR penetrat* OR mechanism* OR mediat* OR driv*** | [7,230,940](https://pubmed-ncbi-nlm-nih-gov.ucc.idm.oclc.org/?term=Feasib%2A%2BOR%2Bsustain%2A%2BOR%2Badopt%2A%2BOR%2Baccept%2A%2BOR%2Bappropriate%2A%2BOR%2Bfidelity%2BOR%2Bimplement%2A%2BOR%2Buptake%2A%2BOR%2Badher%2A%2BOR%2Bfacilitat%2AOR%2Bbarrier%2A%2BOR%2Baccessib%2A%2BOR%2Bpenetrat%2A%2BOR%2Bmechanism%2A%2BOR%2Bmediat%2A%2BOR%2Bdriv%2A&sort&size=100) | 15:05:21 |
| #2 | **…** |  | Search: **(Faller* OR "accidental fall*" OR falling OR falls OR slip* OR "fall prevention" OR "falls prevention") AND (Prevent* OR reduc* OR minimi* OR decreas* OR interven*)** | [58,802](https://pubmed-ncbi-nlm-nih-gov.ucc.idm.oclc.org/?term=%28Faller%2A%2BOR%2B%E2%80%9Caccidental%2Bfall%2A%E2%80%9D%2BOR%2Bfalling%2BOR%2Bfalls%2BOR%2Bslip%2A%2BOR%2B%E2%80%9Cfall%2Bprevention%E2%80%9D%2BOR%2B%E2%80%9Cfalls%2Bprevention%E2%80%9D%29%2BAND%2B%28Prevent%2A%2BOR%2Breduc%2A%2BOR%2Bminimi%2A%2BOR%2Bdecreas%2A%2BOR%2Binterven%2A%29&sort&size=100) | 15:05:00 |
| #1 | **…** |  | Search: **"long-term care setting*" OR "nursing home*" OR**  **"residential care setting*" OR "Residential facilit*" OR "institution care" OR "nursing care" OR "nursing facilit*" OR "continuous care"** | [118,475](https://pubmed-ncbi-nlm-nih-gov.ucc.idm.oclc.org/?term=%E2%80%9Clong-term%2Bcare%2Bsetting%2A%E2%80%9D%2BOR%2B%E2%80%9Cnursing%2Bhome%2A%E2%80%9D%2BOR%2B%E2%80%9Cresidential%2Bcare%2Bsetting%2A%E2%80%9D%2BOR%2B%E2%80%9CResidential%2Bfacilit%2A%E2%80%9D%2BOR%2B%E2%80%9Cinstitution%2Bcare%E2%80%9D%2BOR%2B%E2%80%9Cnursing%2Bcare%E2%80%9D%2BOR%2B%E2%80%9Cnursing%2Bfacilit%2A%E2%80%9D%2BOR%2B%E2%80%9Ccontinuous%2Bcare%E2%80%9D&sort&size=100) | 15:04:28 |
