# Supplementary material for: Implementation strategies to support fall prevention interventions in long-term care facilities for older persons: a systematic review
Source: BMC Geriatr. 2023 Jan 25;23:47. doi: 10.1186/s12877-023-03738-z (PMC9878796; doi:10.1186/s12877-023-03738-z)
Supplement: Supplementary file 2 — Additional file 2: Table 1. The quality appraisal results of included CRCTs (Experimental studies). Table 2. The quality appraisal results of included Quasi-experimental studies. [file 12877_2023_3738_MOESM2_ESM.docx]

| The first author | Q1/Randomization | Q2/Allocation Concealment | Q3/Similar at baseline | Q4/Participants blinded | Q5/Investigators Blinded | Q6/Outcome assessors blinded | Q7/Identical standard care for both groups | Q8/Follow-up | Q9/Analysed in groups | Q10/Outcomes same | Q11/Outcomes reliable | Q12/Appropriate statistical analysis | Q13/Appropriate trial design | The total score = SUM Y/13 |
| --- | --- | --- | --- | --- | --- | --- | --- | --- | --- | --- | --- | --- | --- | --- |
| Colen-Emeric et al. | Y | U | N | N | N | Y | U | Y | Y | Y | Y | Y | Y | 8/13 |
| Kerse et al. | Y | U | N | N | Y | Y | N | Y | Y | Y | Y | Y | Y | 9/13 |
| Ray et al. | Y | Y | N | Y | U | U | N | Y | Y | Y | Y | Y | Y | 9/13 |
| Jensen et al. | Y | Y | Y | N | N | N | N | Y | U | Y | Y | Y | Y | 8/13 |
| Meyer et al. | Y | Y | Y | U | N | N | N | Y | Y | Y | Y | Y | Y | 9/13 |
| Ward et al | Y | U | Y | U | N | N | N | Y | Y | Y | Y | Y | Y | 8/13 |
| Bouwen et al. | Y | U | N | U | U | U | N | Y | Y | N | Y | Y | Y | 6/13 |

**Additional file 2 Table1: The quality appraisal results of included CRCTs (Experimental studies)**

Y: Yes, N: No, U: unclear, NA: Not applicable. (Q1. Was true randomization used for the assignment of participants to treatment groups? Q2. Was allocation to treatment groups concealed? Q3. Were treatment groups similar at the baseline? Q4. Were participants blind to treatment assignment? Q5. Were those delivering treatment blind to treatment assignment? Q6. Were outcomes assessors blind to treatment assignment? Q7. Were treatment groups treated identically other than the intervention of interest? Q8. Was follow-up complete and if not, were differences between groups in terms of their follow-up adequately described and analyzed? Q9. Were participants analyzed in the groups to which they were randomized? Q10. Were outcomes measured in the same way for treatment groups? Q11. Were outcomes measured in a reliable way? Q12. Was appropriate statistical analysis used? Q13. Was the trial design appropriate, and any deviations from the standard RCT design (individual randomization, parallel groups) accounted for in the conduct and analysis of the trial?

**Additional file 2 Table2: The quality appraisal results of included Quasi-experimental studies**

| The first author | Q1/Clear cause and effect | Q2/Similar at baseline | Q3/Identical standard care for both groups | Q4/Control group present | Q5/Multiple measurements | Q6/Follow-up | Q7/Outcomes same | Q8/Outcomes reliable | Q9/Appropriate statistical analysis | The total score=Sum Y/9 |
| --- | --- | --- | --- | --- | --- | --- | --- | --- | --- | --- |
| Jackson | Y | Y | N | N | Y | N | Y | Y | N | 5/9 |
| Zubkoff et al. | Y | Y | N | N | Y | N | Y | Y | Y | 6/9 |
| Leverenz et al. | Y | Y | N | N | N | N | Y | Y | N | 4/9 |
| Rask et al. | Y | N | U | Y | Y | Y | Y | Y | Y | 7/9 |
| Gama et al. | Y | Y | N | N | Y | Y | Y | Y | N | 6/9 |
| Bonner et al. | Y | Y | N | N | Y | N | Y | Y | N | 5/9 |
| Beasley et al. | Y | Y | N | N | Y | N | Y | Y | N | 5/9 |
| Wongrakpanich et al. | Y | Y | N | N | Y | N | Y | Y | N | 5/9 |
| Szczerbinska et al. | Y | Y | N | N | Y | Y | Y | Y | U | 6/9 |
| Copper et al. | Y | Y | N | N | Y | N | Y | Y | N | 5/9 |
| Colon-Emeric et al. | Y | Y | N | Y | Y | N | N | Y | Y | 6/9 |
| Hofmann et al. | Y | U | N | N | Y | U | Y | Y | Y | 5/9 |
| Theodos | Y | Y | U | N | Y | N | Y | Y | Y | 6/9 |
| Kato et al. | Y | Y | N | Y | Y | N | Y | Y | N | 6/9 |
| Wells | Y | y | N | N | Y | Y | Y | Y | N | 6/9 |
| Ofosuhene | Y | Y | N | N | Y | N | Y | Y | N | 5/9 |
| Lomax | Y | Y | N | N | Y | N | Y | Y | N | 5/9 |
| Hurst | Y | Y | N | N | Y | N | Y | Y | N | 5/9 |
| Ogundu | Y | Y | N | N | Y | N | Y | Y | N | 5/9 |
| Aguwa | Y | Y | N | N | N | N | Y | Y | N | 4/9 |

Y: Yes, N: No, U: unclear, NA: Not applicable. (Q1. Is it clear in the study what is the ‘cause’ and what is the ‘effect’ (i.e. there is no confusion about which variable comes first)? Q2. Were the participants included in any comparisons similar? Q3. Were the participants included in any comparisons receiving similar treatment/care, other than the exposure or intervention of interest? Q4. Was there a control group? Q5. Were there multiple measurements of the outcome both pre and post the intervention/exposure? Q6. Was follow up complete and if not, were differences between groups in terms of their follow up adequately described and analyzed? Q7. Were the outcomes of participants included in any comparisons measured in the same way? Q8. Were outcomes measured in a reliable way? Q9. Was appropriate statistical analysis used?
