# Supplementary material for: Implementation strategies to support fall prevention interventions in long-term care facilities for older persons: a systematic review
Source: BMC Geriatr. 2023 Jan 25;23:47. doi: 10.1186/s12877-023-03738-z (PMC9878796; doi:10.1186/s12877-023-03738-z)
Supplement: Supplementary file 3 — Additional file 3. Detailed description of implementation strategies identified. [file 12877_2023_3738_MOESM3_ESM.docx]

**Additional file 3: Detailed description of implementation strategies identified**

| Author | **Train and educate stakeholders** | **Develop stakeholder interrelationships** | **Use evaluative and iterative strategies** | **Provide interactive assistance** | **Support clinicians** | **Adopt and tailor to context** | **Change infrastructure** | **Utilise financial strategies** | **Engage consumers** |
| --- | --- | --- | --- | --- | --- | --- | --- | --- | --- |
| Bonner et al., | **In-service/once-off education**: 40 mins/twice per shift | - **Organise clinician meetings:** arranged time and day for presentation - **Champion** | **Purposefully re-examine implementations**: weekly discussions to track issues from staff rounds related to a safe environment, skills employed and equipment | - **Facilitation:** ongoing support provided to staff participating in the programme - **Clinical supervision:** weekly interdisciplinary rounds with facility staff, monitoring transfer techniques | **Remind clinicians:**  "Quick Tips" badge to be worn by all staff, with each staff using a checklist to check before leaving residents’ rooms. | **Tailor strategy:** The housekeeping supervisor provided translations for staff who spoke Spanish | **Change physical structure and equipment:** laundry receptacles in hallways and the use and storage of housekeeping and maintenance equipment | **Alter incentive/ allowance:** Staff were compensated for their attendance on a day off by the institution. |  |
| Gama et al., | **Once-off education:** 5 * 1-hour multidisciplinary sessions |  |  | **Facilitation:** Bi-monthly meetings to solve problems | - **Remind clinicians:** a reminder in the falls registry system indicating the need for a medical assessment after falls - **Revise professionals' roles:** implementation of a revised fall risk management guideline outlining each professional's responsibilities |  | - **Change record system:** changes in registration systems (assessment tools) - **Change physical structure and equipment:** The location of the falls registry was changed. |  |  |
| Leverenz et al. | - **In-service/ongoing education:** weekly group sessions conducted by OT - **Develop educational materials**: 6 learning modules; different content areas for preventing falls - **Distribute educational materials:** online and via notebooks. - **Ongoing training:** weekly individual collaborative session facilitated by OT (5 wks.) - **Ongoing consultations:** dialogue provided by OT about the fall prevention strategies as related to the immediate resident-care context - **Make education dynamic**: acronyms, alliteration, visual representations and experiential learning activities - **Make training dynamic:** one-to-one collaborations between nursing staff members and educators |  |  | **Facilitation:** communication with nurses about their ability to recognise and implement various fall prevention strategies |  |  |  |  |  |
| Rask et al., | **Develop and distribute educational materials**: a manual; companion Living Space Inspection notebook; a video; laminated brochures; appendices included all forms; a case history with examples; all resources; formal communication tool   - **Ongoing training**: a one-day workshop per month - **Ongoing consultations:** two advanced practice nurses providing consultation by telephone about fall intervention strategies - **Make training dynamic:** problem-solving skills; discussion of a new intervention for recurrent fallers | - **Champions** - **Leaderships** - **Build a coalition:** explain the intervention program for the corporate officers of facilities - **Use advisory board and groups:** each facility selected FT | - **Develop and implement tools:** the Tracking Record for Improving Patient Safety (TRIPS) and flow chart - **Develop and organise quality monitoring systems:** a companion Web-based software programme - **Local needs assessment:** self-assessment to identify weaknesses in fall teams - **Purposefully re-examine implementations:** facilities staff reported success and barriers | - **Facilitation:** APN support staff via monthly teleconference discussions - **Clinical supervision:** Two corporate officers and regional nurses supervised one or more facilities. |  | **Promote adaptability:** Physicians chose not to use the facsimile system instead of relying on pre-established telephone communication or weekly visits. | **Change record system:** reduced repetitive and lengthy documentation |  |  |
| Jackson et al., | **Once-off training:** mobility training (transfer, gait training, muscle strengthening and balance exercises) and environmental assessment | - **Use advisory board and workgroups:** selected FT (10 staff) - **Organise clinician meetings:** Monthly meeting held | - **Develop and implement tools:** staff rounding notation sheets - **Develop and organise quality monitoring systems:** every two-hour rounding and documentation of rounding | **Facilitation:** Organisation-wide coaching to ensure staff understood their roles in patient safety and their adherence to fall prevention initiatives |  | **Promote adaptability:** Restorative care physical activities were provided for residents not involved in a regular physical therapy regimen |  |  |  |
| Szczerbinksa et al., | **Once-off training:** (3 hours) introduction to fall risks and fall-related injuries, demonstration of TUG and POMA tests; exercise therapy for high-fall-risk residents |  | **Develop and implement tools:** the falls registration form | - **Clinical supervision:** PT supervised the exercise interventions. - **Centralise technical assistance**: weekly visits to provide advice and assistance in filling out specially developed forms |  |  |  |  |  |
| Colon-Emeric et al., A | - **Ongoing/in-service/online education:** 2 in-person sessions + 5 teleconferences over 9 months - **Develop and distribute educational materials:** A toolkit of customisable forms, stickers, posters and educational materials | **Use advisory broad and workgroups:** quality improvement teams |  |  | **Remind clinicians:** "labelling" high-risk residents with chart, room and armband stickers |  |  |  |  |
| Wongrakpanich et al., | - **Ongoing training:** 1 * 3 implementation checklists per month - **Distribute educational materials:** STEADI patient and family caregiver brochures | **Organise clinical meeting:** Monthly meeting held | **Develop and implement a tool for quality monitoring:** The checklist using a colour-coding system |  |  |  |  |  | **Involve patients and family caregivers:** family caregiver in education |
| Cooper | **Once-off training**: Introduced the Falls Risk and intervention tool. | - **Organise clinical implementation meeting:** Staff huddles gathered 5-15 minutes on night shift - **Champions** | - **Develop and implement tools:** a chart for recorded falls - **Conduct cyclical small test** - **Local needs assessment:** | - **Facilitation:** Test 1:1 handover with demonstrations or photographs to ensure clarity and compliance with implementation. **Local technical assistance:** A registered nurse was identiﬁed to support them with the tests. |  |  | **Start a dissemination organisation:** Two senior care assistants & two nurses took responsibility for initiating huddles in the facility. |  | **Involve patients and family caregivers:** family involved in root cause analysis exercises |
| Zubkoff et al. | **Ongoing/online education:** 2 calls per month (during 3 months); one an educational call, the other a discussion call | - **Capture and share local knowledge**: participants shared details on specific topics - **Champions** - **Leaderships** | - **Assess for readiness:** a "meet and greet" call to identify team aims and problems each team was facing - **Audit/feedback** - **Local needs assessment:** Baseline data of current fall prevention efforts - **Purposefully re-examine implementation:** submitted reports at the end of each phase | **Local technical assistance:** Coaches were available for assistance as needed.  **Centralised technical assistance:** "open discussion" call to answer questions and troubleshoot | **Create clinician teams:** sites developed teams to complete the programme application |  |  |  |  |
| Beasley | - **Ongoing/in-service education:** 3 sessions provided by PT and team leader; ongoing annual sessions - **Distribute educational materials:** The JBI Best Practice Information Sheet on fall prevention | **Designate leaderships** | - **Audit/feedback** - **Local needs assessment:** baseline audit and reviewing of the current policies of facilities - **Purposefully re-examine implementation:** reassess the programme and reassess the cause of falling after residents fall |  | **Remind clinicians:** highlight those residents assessed as being at a high risk of falling on the resident handover sheets, as a daily reminder |  |  |  |  |
| Hofmann et al., |  | **Use advisory broad and workgroups:** a multidisciplinary committee to address the increase in falls | **Develop and organise quality monitoring** **systems:** computerised quality indicators | **Local technical assistance:** Furniture was repositioned with the assistance of occupational therapy staff to allow for more manoeuvring space. |  |  |  |  |  |
| Theodos | **Ongoing training:** quarterly instruction of sit-to-stand exercise, positioning, transfer for all staff  **Distribute and develop educational materials:** a video, poster and brochure for exercises; listing of suggested case management/nursing interventions for fall-risk residents | **Use advisory board and work groups:** a multidisciplinary safety committee to make monthly safety round and environmental hazard assessments | - **Audit/feedback** - **Develop and implement a tool for quality monitoring**: the Monitor Tool; post-fall assessment tools |  | - **Remind clinicians:** blue dots on medical records, armbands (when one falls, they turn black and blue); posters were placed in key locations. **Create clinician teams:** teams selected by the case manager |  |  |  |  |
| Kato et al., | - **Ongoing Education:** 2 seminars over six months - **Ongoing clinical consultation:** fall teams and medical staff offered to provide information about fall-related problems or concerns | - **Use advisory boards and workgroups:** each facility selected FT - **Organise clinician meetings**: Monthly meeting held | **Audit** |  |  | **Promote adaptability**: Based on the assessment, care was adapted to the risks and was chosen based on safety and comfort. |  |  |  |
| Jensen et al. | **Once-off/in-service education:** 4-hour educational session facilitated by a physician and PT | **Organise clinician meetings:** Weekly meeting held | **Develop and implement tools for monitoring:** fall report forms |  |  |  |  |  |  |
| Kerse et al., | - **Once-off/in-service education:** 2 * 1-hour sessions for RN and assistant nurses in each home. - **Distribute and develop educational materials:** manual containing the risk assessment form, information for strategies, high-risk fall logos, all forms and educational information for different disciplines. | **Leaderships** | **Audit** | **Facilitation:** visits to ensure fall coordinators understood the risk assessment process | **Remind clinicians:** high-risk logo, including colour-coded dots, attached to the walls of the rooms of residents at a high risk of falling |  |  |  |  |
| Ray et al. | - **Once-off training:** 2-day regional workshop on patient safety   **Make training dynamic:** lectures, group discussions and hands-on sessions   - **Distribute and develop educational materials:** a manual, video, training materials and assessment forms | **Leaderships** |  | **Facilitation:** phone calls to solve implementation issues and assist with resident assessment and treatment planning | Create a clinician team |  |  |  |  |
| Meyer et al. | - **Once-off/in-service education:** 60–90-min session provided by a researcher - **Develop and distribute educational materials**: Structured evidence-based fall prevention strategies for nursing staff, brochures - **Make training dynamic:** presentation, group work and plenary discussion, ﬁctitious vignettes |  |  |  |  |  |  |  |  |
| Colon-Emeric et al., b | - **Ongoing/in-service education:** 2 * 1-hour learning sessions per month - **Distribute educational materials:** Online and paper form - **Develop educational materials:** 3 self-study case-based modules - **Make training dynamic:** Storytelling and role play - **Ongoing training:** 3 sessions per month | **Promote networking weaving:** improved group-to-group interaction patterns  **Organise clinician meetings:** Weekly teleconferences meeting | **Audit/feedback** | **Facilitation:** managing communication problems via brief discussions with each individual |  |  |  |  |  |
| Ward et al. | - **Ongoing training:** Workshops on implementing evidence-based fall interventions, particularly exercises - **Create collaborative learning** - **Distribute and develop educational materials:** provide a set of fall prevention resources and website links |  | **Develop and implement tools for quality monitoring:** a standard form for collecting details regarding the number of falls | **Facilitation:** employment of a project nurse to encourage a range of best practice strategies at the facilities  **(facilitator)** |  |  | **Mandate change:** Divisions of general practice supported for intervention |  |  |
| Bouwen et al., | - **Once-off training:** Fall risk, environmental modifications - **Develop and distribute educational materials:** organised for low-staff occupations and received presentations copies |  | **Develop and implement tools for quality monitoring:** Nurses kept a fall diary |  | **Remind clinicians:** no details |  |  |  |  |
| Lomax | - **In-service/once-off education:** presentation about post-fall huddles and fall preventions, provided by a researcher   **Distribute educational materials:** Educational information for post-fall huddles from the National Falls Toolkit designed by the United States Department of Veteran Affairs (2014). | **leaderships** | **Develop and organise quality monitoring systems (procedures):** completion of the Post-Fall Huddle Form, attaching it to the incident report |  |  |  |  |  |  |
| Wells | - **In-service/ongoing education:**1 * 3 sessions per month, 30-45 mins for each class - **Develop and distribute educational materials**: a syllabus for a fall prevention course, involving all outline information - **Make education dynamic:** visual activities (pictures, videos, PowerPoint presentations); auditory (audio recording of lecture) - **Make training dynamic:** kinaesthetic activities (interactive learning and teamwork, and critical thinking activities based on learners' experiences) | - **Capture and share local knowledge:** participants shared their experiences and techniques to handle falls - **Champions** |  | **Facilitation:** assisting CNAs in developing a personal interest and motivating them to develop a personal desire to prevent falls |  |  |  |  |  |
| Ofosuhene | - **In-service/once-off education:** 1-hour sessions per shift using STEADI core elements, facilitated by the investigator - **Make education dynamic:** Presentation: 20 mins, video: 15 mins, discussion and practice: 10 mins | **Champions** | **Purposefully re-examine implementation:** stakeholders met to monitor the post-implementation progress with STEADI |  |  |  |  |  |  |
| Hurst | - **In-service/once-off education:** mandatory presentation for all staff (three shifts) - **Distribute and develop educational materials:** PowerPoint presentation of fall assessment, utilise Electronic Morse Falls Risk Scale and disseminate pertinent information to the staff involved in daily care | - **Champions** - **Use advisory board and groups:** developed FT | - **Develop and implement tools:** the data collection tool was utilised to capture the weekly fall rates - **Develop and organise quality monitoring systems**: fall champions kept track of weekly fall numbers. - **Purposefully re-examine implementations:** falls champions reported to FT for reviews and updates |  |  |  |  |  |  |
| Ogundu | - **Once-off education:** teaching about hourly rounding, the definition of the 4 Ps in hourly rounding and documentation   **Distribute and develop educational materials:**  educational content of fall prevention and hourly rounding with providing all recourses of evidence | **Identified champions** | **Develop and implement tools for quality monitoring:** the hourly rounding log |  |  |  |  |  |  |
| Aguwa | - **In-service/once-off education**: introduced AMDA's fall prevention guideline and the STEADI toolkit - **Distribute educational materials:** The STEADI toolkit involves an educational video and handout. |  |  |  | **Create a clinician team:** The clinical nurse leader formed a clinician team of three nurses. |  |  |  |  |

**OT:** occupational therapist; **PT**: physical therapist; **Wks**: weeks; **FT**: fall teams; **TUG**: Time-Up-Go test; **POMA**: modified Tinetti Performance Oriented Mobility Assessment; **STEADI:** Stopping Elderly Accidents, Death and Injuries; **ADMA:** American Medical Directors Association Guideline
