# Supplementary material for: Implementation strategies to support fall prevention interventions in long-term care facilities for older persons: a systematic review
Source: BMC Geriatr. 2023 Jan 25;23:47. doi: 10.1186/s12877-023-03738-z (PMC9878796; doi:10.1186/s12877-023-03738-z)
Supplement: Supplementary file 4 — Additional file 4. The codebook definitions of implementation strategies identified. [file 12877_2023_3738_MOESM4_ESM.docx]

**Additional file 4: The codebook definitions of implementation strategies identified**

| Name of strategy | | Definition from ERIC list (Waltz et al., 2015)(38) |
| --- | --- | --- |
| Promote adaptability | | Identify the ways a clinical innovation can be tailored to meet local needs and clarify which elements of the innovation must be maintained to preserve fidelity |
| Tailor strategies | | Tailor the implementation strategies to address barriers and leverage facilitators that were identified through earlier data collection. |
| Change physical structure and equipment | | Evaluate current configurations and adapt, as needed, the physical structure and/or equipment (e.g., changing the layout of a room, adding equipment) to best accommodate the targeted innovation |
| Change record systems | | Change records systems to allow better assessment of implementation or clinical outcomes |
| Mandate change | | Have leadership declare the priority of the innovation and their determination to have it implemented. |
| Start a dissemination organization | | Identify or start a separate organization that is responsible for disseminating the clinical innovation. It could be a for-profit or non-profit organization. |
| Build a coalition | | Recruit and cultivate relationships with partners in the implementation effort. |
| Capture and share local knowledge | | Capture local knowledge from implementation sites on how implementers and clinicians made something work in their setting and then share it with other sites. |
| Identify and prepare champions | | Identify and prepare individuals who dedicate themselves to supporting, marketing, and driving through an implementation, overcoming indifference or resistance that the intervention may provoke in an organization |
| Organise clinical implementation team meetings | | Develop and support teams of clinicians who are implementing the innovation and give them protected time to reflect on the implementation effort, share lessons learned, and support one another’s learning. |
| Promote network weaving | | Identify and build on existing high quality working relationships and networks within and outside the organization, organizational units, teams, etc. to promote information sharing, collaborative problem-solving, and a shared vision/goal related to implementing the innovation. |
| Recruit, designate, and train for leadership | | Recruit, designate, and train leaders for the change effort. |
| Use advisory boards and workgroups | | Create and engage a formal group of multiple kinds of stakeholders to provide input and advice on implementation efforts and to elicit recommendations for improvements. |
| Involve patients and family caregivers | | Engage or include patients/consumers and families in the implementation effort. |
| Centralize technical assistance | | Develop and use a centralized system to deliver technical assistance focused on implementation issues |
| Facilitation | | A process of interactive problem solving and support that occurs in a context of a recognized need for improvement and a supportive interpersonal relationship |
| Provide clinical supervision | | Provide clinicians with ongoing supervision focusing on the innovation. Provide training for clinical supervisors who will supervise clinicians who provide the innovation. |
| Provide local technical assistance | | Develop and use a system to deliver technical assistance focused on implementation issues using local personnel. |
| Create new clinician teams | | Change who serves on the clinical team, adding different disciplines and different skills to make it more likely that the clinical innovation is delivered (or is more successfully delivered). |
| Remind clinicians | | Develop reminder systems designed to help clinicians to recall information and/or prompt them to use the clinical innovation |
| Revise the professional roles | | Shift and revise roles among professionals who provide care, and redesign job characteristics |
| **Conduct educational outreach visits/ educational meetings** | | **Educational outreach visits**: Have a trained person meet with providers in their practice settings to educate providers about the clinical innovation with the intent of changing the provider’s practice. Visits to the site may be in-person or virtually via the Internet. Some initiatives may require regular educational outreach as part of maintaining the innovation/practice change. **Educational meetings:** Hold meetings targeted toward different stakeholder groups (e.g., providers, administrators, other organizational stakeholders, and community, patient/consumer, and family stakeholders) to teach them about the clinical innovation. |
| ***The frequency of education program*** | Once-off education | Educate the stakeholders about the clinical innovation once, considering that the duration of the program ranges from hours to several days. |
|  | Ongoing education | Educate the stakeholders about the clinical innovation ongoing way (monthly, annually) |
| ***The mode of education delivery*** | In-service education sessions | Educate stakeholders about the clinical innovation in their site (in-service) face to face. |
|  | Online education sessions | Educate stakeholders about clinical innovation virtually via the Internet. |
| ***Conduct once-off training*** | | conduct training for targeting groups about clinical innovations once only. |
| Conduct ongoing training | | Plan for and conduct training in clinical innovation in an ongoing way. |
| Create a learning collaborative | | Facilitate the formation of groups of providers or provider organizations and foster a collaborative learning environment to improve implementation of the clinical innovation |
| Develop educational materials | | Develop and format manuals, toolkits, and other supporting materials in ways that make it easier for stakeholders to learn about the innovation and for clinicians to learn how to deliver the clinical innovation. |
| Distribute educational materials | | Distribute educational materials (including guidelines, manuals and toolkits) in person, by mail, and/or electronically |
| ***Make education dynamic*** | | Using different educational delivery methods to deliver the educational contents that serve the educational content to be more interactive for learners |
| Make training dynamic | | Vary the information delivery methods to cater to different learning styles work contexts, and shape the training in the innovation to be interactive |
| Provide ongoing consultation | | Provide ongoing consultation with one or more experts in the strategies used to support implementing the innovation |
| ***Provide ongoing clinical consultation*** | | Provide ongoing consultation with one or more experts in the clinical problem solving |
| Assess for readiness | | Assess various aspects of an organization to determine its degree of readiness to implement, barriers that may impede implementation, and strengths that can be used in the implementation effort. |
| Audit and provide feedback | | Collect and summarize clinical performance data over a specified time period and give it to clinicians and administrators to monitor, evaluate, and modify provider behaviour. |
| Conduct cyclical small tests of change | | Implement changes in a cyclical fashion using small tests of change before taking changes system-wide. Tests of change benefit from systematic measurement, and results of the tests of change are studied for insights on how to do better. This process continues serially over time, and refinement is added with each cycle. |
| Conduct local needs assessment | | Collect and analyse data related to the need for the innovation |
| Develop and implement tools for quality monitoring | | Develop, test, and introduce into quality-monitoring systems the right input—the appropriate language, protocols, algorithms, standards, and measures (of processes, patient/consumer outcomes, and implementation outcomes) that are often specific to the innovation being implemented |
| Develop and organize quality monitoring system | | Develop and organize systems and procedures that monitor clinical processes and/or outcomes for the purpose of quality assurance and improvement |
| purposefully re-examine the implementation | | Monitor progress and adjust clinical practices and implementation strategies to continuously improve the quality of care. |
| Alter incentive-allowance structures | | Work to incentivize the adoption and implementation of the clinical innovation |
