# Supplementary material for: Implementation strategies to support fall prevention interventions in long-term care facilities for older persons: a systematic review
Source: BMC Geriatr. 2023 Jan 25;23:47. doi: 10.1186/s12877-023-03738-z (PMC9878796; doi:10.1186/s12877-023-03738-z)
Supplement: Supplementary file 5 — Additional file 5. Detailed description of the reasons for excluding papers. [file 12877_2023_3738_MOESM5_ESM.docx]

Additional file 5: Detailed description of the reasons for excluding papers

| Inclusion criteria | Additional information on reasons for exclusion |
| --- | --- |
| Study targeted:   - **All staff members** - In **LTCFs** - Working with **older people** (aged 65 and above) | Incorrect resident population:   - Age: mixed age population of adults/older adults (without separation of data); or only people under 65 years; or population was 50 years or older, or 60 years or older (n=23). - Specialised facilities: mental health facilities, and units exclusively for people with cognitive issues, intellectual disability, physical disabilities, etc., without data separation (n=7). - Sub-population: The focus of the intervention was directed only at a specific population within a LTCF, such as those with joint contracture, dementia, and stroke (n=7). |
|  | Incorrect setting:   - Acute settings (n=9) - Community settings (n=11) - Assisted living settings (n=6) - Mixed incorrect settings (community, assisted living setting) (n=2) - Short-stay (transitional unit) (n=1). |
|  | Mixed population setting (community and long-term care facilities) data not separated (n=5). |
|  | Not targeting staff:   - Targeting residents and families (i.e., exercise interventions without involving staff) (n=10). - Nursing students only, not the LTCF staff (n=2). |
| **Fall prevention** **interventions**, whether a single-component or multifactorial /multicomponent intervention, where there was an **implementation strategy** or **implementation process** described.  Intervention | Non-experimental study design:   - Longitudinal naturalistic studies (retrospective/prospective) (n=12) - Cross-sectional studies (n=3); - Qualitative studies (n=8). |
|  | Intervention was not focused on falls prevention:   - Improving documentation/reporting of fall incidents only (n=7) - Improving medication prescribing (n=12) - Introducing a model of care or technology (n=5) - Vitamin D supplementation (n=3) - Reducing hip fractures (via other means than fall prevention) (n=12) - Reducing bed rail use (n=3) - Reducing inappropriate transfers to hospitals (n=1) |
| Full paper available | - Conference abstract only (n=8) or thesis abstract only (n=6) |
| Non-English/Arabic language | One article was published in German |
